# DEK-targeting DNA aptamers as therapeutics for inflammatory arthritis

**DOI:** 10.1038/ncomms14252

**Published:** 2017-02-06

**Authors:** Nirit Mor-Vaknin, Anjan Saha, Maureen Legendre, Carmelo Carmona-Rivera, M Asif Amin, Bradley J. Rabquer, Marta J. Gonzales-Hernandez, Julie Jorns, Smriti Mohan, Srilakshmi Yalavarthi, Dave A. Pai, Kristine Angevine, Shelley J. Almburg, Jason S. Knight, Barbara S. Adams, Alisa E. Koch, David A. Fox, David R. Engelke, Mariana J. Kaplan, David M. Markovitz

**Affiliations:** 1 Department of Internal Medicine, Division of Infectious Diseases, University of Michigan, Ann Arbor, Michigan 48109, USA; 2 Program in Cancer Biology, University of Michigan, Ann Arbor, Michigan 48109, USA; 3 Systemic Autoimmunity Branch, National Institute of Arthritis and Musculoskeletal and Skin Diseases, Bethesda, Maryland 20892, USA; 4 Department of Internal Medicine, Division of Rheumatology, University of Michigan, Ann Arbor, Michigan 48109, USA; 5 Department of Pathology, University of Michigan, Ann Arbor, Michigan 48109, USA; 6 Department of Pediatrics and Communicable Diseases, Division of Pediatric Rheumatology, University of Michigan, Ann Arbor, Michigan 48109, USA; 7 Department of Biological Chemistry, University of Michigan, Ann Arbor, Michigan 48109, USA; 8 Microscopy & Image – Analysis Laboratory, University of Michigan, Ann Arbor, Michigan 48109, USA; 9 VA Medical Service, Department of Internal Medicine/Division of Rheumatology, University of Michigan, Ann Arbor, Michigan 48105, USA; 10 Programs in Immunology, Cellular & Molecular Biology, and Cancer Biology, University of Michigan, Ann Arbor, Michigan 48109, USA; 12 Deceased

## Abstract

Novel therapeutics are required for improving the management of chronic inflammatory diseases. Aptamers are single-stranded RNA or DNA molecules that have recently shown utility in a clinical setting, as they can specifically neutralize biomedically relevant proteins, particularly cell surface and extracellular proteins. The nuclear chromatin protein DEK is a secreted chemoattractant that is abundant in the synovia of patients with juvenile idiopathic arthritis (JIA). Here, we show that DEK is crucial to the development of arthritis in mouse models, thus making it an appropriate target for aptamer-based therapy. Genetic depletion of DEK or treatment with DEK-targeted aptamers significantly reduces joint inflammation *in vivo* and greatly impairs the ability of neutrophils to form neutrophil extracellular traps (NETs). DEK is detected in spontaneously forming NETs from JIA patient synovial neutrophils, and DEK-targeted aptamers reduce NET formation. DEK is thus key to joint inflammation, and anti-DEK aptamers hold promise for the treatment of JIA and other types of arthritis.

## Abstract (web summary)

DEK is a secreted protein abundant in the synovia of patients with juvenile idiopathic arthritis. Here the authors show DEK is important for neutrophil extracellular trap formation and joint inflammation, and demonstrate therapeutic efficacy of DEK-targeting aptamers in a mouse model of arthritis.

Inflammatory arthritis causes substantial disability in adults and children. While diagnosis and treatment have advanced considerably over recent years due to the introduction of anti-cytokine therapies, including tumour necrosis factor (TNF) inhibitors and, more recently, inhibitors of interleukin (IL)-1 and IL-6 (ref. 1), these therapies can lead to opportunistic infections, are extremely expensive and can have long-term side effects. Mechanistic insight into the chronic joint inflammation characteristic of rheumatoid arthritis (RA) and juvenile idiopathic arthritis (JIA) is severely lacking, warranting a need for identifying novel targets that carry therapeutic promise.

An attractive therapeutic avenue involves the use of aptamers, which are single-stranded DNA or RNA oligonucleotides that can be designed to specifically target and inactivate clinically relevant molecules. Aptamers are generated through a process termed Systematic Evolution of Ligands by Exponential Enrichment (SELEX), whereby high-affinity candidates targeting a protein of interest are selected from a pool of random-sequence oligonucleotides. Cell surface and extracellular proteins are particularly favourable targets for aptamers. In fact, an aptamer that targets the pro-angiogenic molecule vascular endothelial growth factor has been approved for the treatment of macular degeneration^2^,^3^,^4^. Identifying and targeting molecules that are considered crucial drivers of the pathogenesis of RA and JIA with aptamers may thus offer an alternative strategy for treating these debilitating chronic diseases.

An example of one such potential target is the nuclear auto-antigen DEK. While its endogenous functions primarily concern chromatin architecture and gene regulation, we have previously shown that DEK is actively secreted by human macrophages and passively released by apoptotic T cells, with subsequent chemoattractant properties^5^,^6^. We also demonstrated that DEK is not only secreted, but can enter neighbouring cells by a heparan-sulfate peptidoglycan-dependent pathway and correct the global heterochromatin and DNA repair defects seen in DEK knockdown cells^7^,^8^. Circulating autoantibodies against DEK have been identified in JIA patients^9^,^10^,^11^,^12^. Importantly, DEK and DEK auto-antibodies are abundant in synovial fluids (SFs) of JIA patients, with a propensity to form intra-articular immune complexes^5^. It is thus conceivable that DEK plays a central role in the pathogenesis of JIA, making it a potentially important therapeutic target. Proof of a direct role for DEK in inflammation has, however, been lacking.

We demonstrate here that genetic depletion and aptamer-mediated targeting of DEK confers protection against arthritis in a murine model of inflammatory arthritis. Mechanistic studies reveal that DEK is crucial to the formation of neutrophil extracellular traps (NETs), structures composed of DNA, histones and antimicrobial factors that have been reported to play a part in the pathogenesis of inflammatory and autoimmune diseases, including RA (refs 13, 14, 15). As DEK-targeting aptamers reduce NET formation in zymosan-injected joints and human peripheral blood neutrophils, we conclude that targeting DEK in the setting of arthritis, especially with aptamers, may serve as a viable therapeutic strategy.

## Results

### Zymosan induces less joint inflammation in *Dek*-KO mice

Intra-articular (i.a.) injection of zymosan, a polysaccharide that is composed primarily of glucan and mannan residues from the cell wall of *Saccharomyces cervesiae*^16^, induces inflammatory arthritis in mice^17^. Cell types that recognize and ingest zymosan, including monocytes, macrophages and neutrophils, become activated following this ingestion. We thus utilized the ZIA murine model to evaluate the local effect of DEK in joint inflammation.

**Figure 1:**
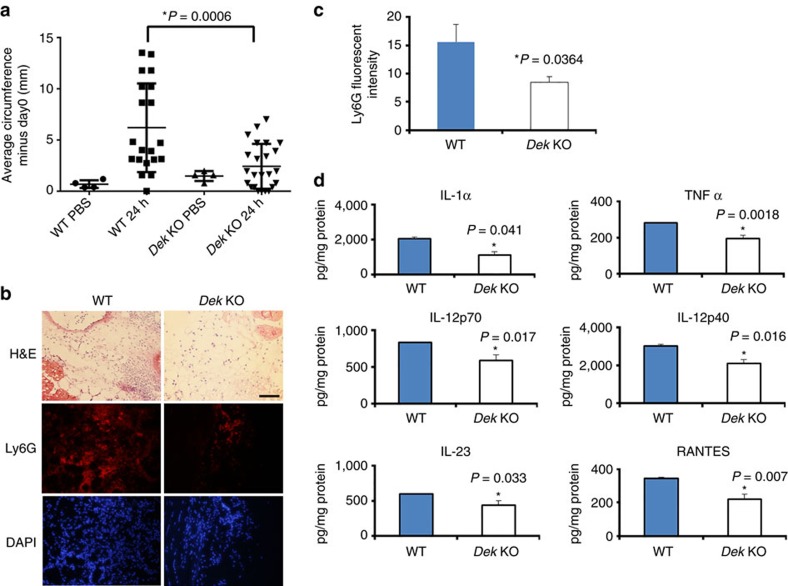
Zymosan induction of joint inflammation is impaired in *Dek*-KO mice. (**a**) WT and *Dek*-KO mice were injected on day 0 with PBS or zymosan. Circumferences were measured at time of injection (day 0), as well as at 24 h after injection. Mean values of the increase in knee circumference are shown at 24 h after injection. The difference in the circumference at 24 h between WT and *Dek*-KO mice was statistically significant (*P=0.0006), as determined by two-tailed, unpaired Student's *t*-test (error bars, s.e.m.). Results shown are for individual mice and the average of four WT versus four *Dek*-KO for PBS control and 20 WT versus 23 *Dek*-KO mice for 24 h after zymosan injection (results shown are from four independent experiments). (**b**) Representative hematoxylin and eosin staining of knee joint sections of three WT and three *Dek*-KO mice 24 h after zymosan injection are shown in the top panel. In the middle panel, joint sections were analysed for neutrophils by immunohistochemistry using the murine neutrophil surface marker Ly6G (in red) 24 h after intra-articular injection. Sections stained for cell nuclei with DAPI (blue) are shown in the lower panel. Magnification × 40 (scale bar 100 μm). (**c**) Arbitrary fluorescent intensity of Ly6G staining was analysed for three different fields of each section of six WT and five *Dek*-KO zymosan-injected knees (*P=0.0364), as determined by two-tailed, unpaired Student's *t*-test (error bars, s.e.m.). Mean fluorescent intensity of the whole section was determined by Image J. (**d**) Inflammatory cytokine profile of knee homogenates 24 h after zymosan injection, as detected by ELISA. Cytokine levels were normalized by protein concentration and were significantly lower in *Dek*-KO versus WT knee homogenates (error bars, s.e.m.). Results shown are average cytokine levels from 7 WT and 11 *Dek*-KO mice.

WT mouse knees were at least two-fold larger (3.794 mm^3^±0.412) than *Dek*-KO mouse knees (1.689 mm^3^±0.282) 24 h following zymosan injection (Fig. 1a; *P*=0.0006, as determined by two-tailed, unpaired Student's *t*-test). Histopathologic analysis of total inflammatory cell infiltration by hematoxylin and eosin staining of knee sections 24 h post-injection demonstrated modest differences between *Dek*-KO and WT mice (Fig. 1b and Supplementary Fig. 1A). Immunostaining for the myeloid marker CD11b yielded no differences (Supplementary Fig. 1B). However, staining for the neutrophil marker Ly6G revealed a significantly dampened neutrophil response in *Dek*-KO mice as compared to WT mice (Fig. 1b,c). Previous reports have demonstrated that aberrant granulocyte differentiation results from DEK knockdown in CD34+ human bone marrow cells^18^. We thus considered that altered neutrophil development might contribute to the milder neutrophil-specific response exhibited by *Dek*-KO mice. Bone marrow and peripheral blood neutrophil counts by Ly6G staining, however, displayed no differences between *Dek*-KO and WT mice (Supplementary Fig. 2A–E).

To further investigate the mechanism behind the reduced inflammatory response in *Dek*-KO mice, we quantified inflammatory cytokines within knee homogenates. Levels of IL-1α, TNF-α, IL-12p40, IL-12p70, IL-23, and regulated on activation normal T cell expressed and secreted (RANTES) were all significantly reduced in knee homogenates from *Dek*-KO mice 24 h post-injection as compared to WT counterparts (Fig. 1d). In contrast, several other inflammatory cytokines did not display significant differences (Supplementary Fig. 3A). To test if the difference in inflammatory responses between WT and *Dek*-KO mice is due to differences in cell signalling, we first analysed expression levels of the cell surface receptor for zymosan, toll-like receptor 2 (TLR2). TLR2 mRNA and protein levels in knee homogenates and cells purified from WT spleens did not differ from expression detected in *Dek*-KO mice (Supplementary Fig. 3B). Furthermore, key regulators of inflammatory cytokines such as NF-κB (pIKKα), MAPK (p38) and pERK1/2 were not differentially regulated in *Dek*-KO versus WT bone marrow macrophages and neutrophils upon *in vitro* stimulation with lipopolysaccharide (LPS) or zymosan (Supplementary Fig. 4). In summary, *Dek*-KO mice develop significantly less inflammation as compared to WT mice in the setting of ZIA as measured by marked changes in joint circumference and cytokine profile, and a modest decrease in neutrophil migration. These effects are not due to obvious differences in neutrophil development or cell signalling events.

### DEK targeting aptamers reduce inflammation in ZIA

**Figure 2:**
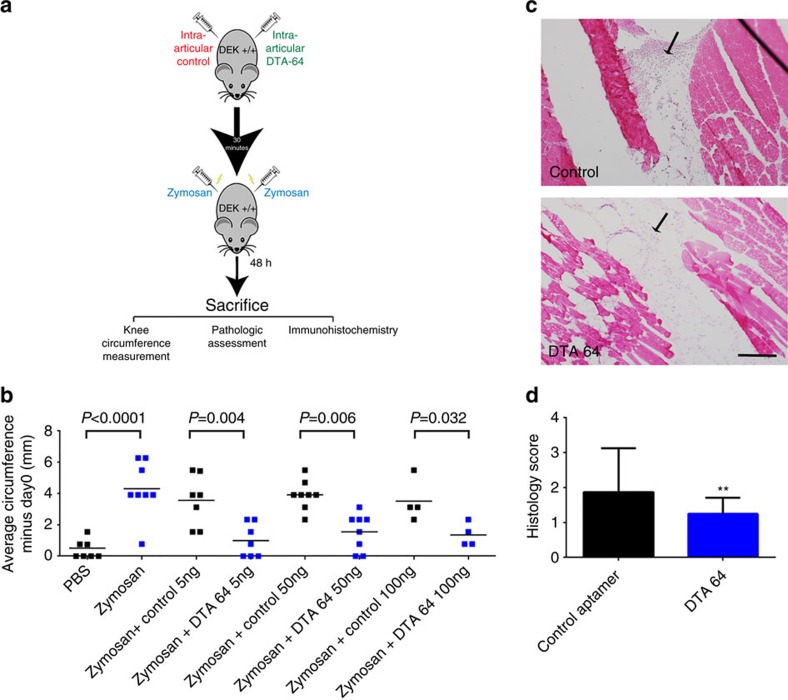
Zymosan induction of joint inflammation is blocked by DEK aptamers. (**a**)WT mice were injected on day 0 with 5, 50 or 100 ng per knee of non-specific DNA aptamer controls or DEK-specific aptamer (DTA-64) prior to injection with PBS alone or zymosan alone as negative and positive controls, respectively. Knee circumferences were measured at the time of injection (day 0), as well as at 48 h following injection. (**b**) Mean values of increased knee circumferences are shown at 48 h after injection. Results shown are from 4–8 individual mice per group and from two independent experiments (*n*=53 mice). Differences in the knee circumference between mice receiving control aptamers versus DTA 64 aptamer (5–100 ng) were statistically significant (*P<0.032) as determined by two-tailed, unequal variance Student's *t*-test. (**c**) Representative sections from seven knees injected with 50 ng control aptamer versus seven knees injected with 50 ng DTA-64 stained by H&E at 48 h after injection. Arrowheads indicate inflammatory cell migration into the knee joint. Magnification × 10 (scale bar 200 μm). (**d**) Semi-quantitative scoring of blinded histological assessment from control aptamer-injected knees (*n*=8) or DTA-64-injected knees (*n*=8) at 50 ng aptamer per knee (**P=0.0056) as determined by two-tailed, unequal variance Student's *t*-test. Results are from two independent experiments (error bars, s.e.m.).

We next explored the possibility that pharmacologic inhibition of DEK could phenocopy genetic depletion in mice. We thus generated anti-DEK aptamers using SELEX technology and selected one single-stranded DNA aptamer, with a 41 nucleotide core with high affinity for recombinant DEK protein (Supplementary Fig. 5). To test its anti-inflammatory capabilities *in vivo*, we injected it into the knee joint 30 min prior to administration of zymosan; the contralateral knee of each mouse was injected with a control aptamer, as illustrated in Fig. 2a. Indeed, our lead candidate DEK-targeting aptamer #64 (DTA-64) significantly reduced joint inflammation at a concentration of 5 ng knee^−1^ (*P*=0.004), 50 ng knee^−1^ (*P*=0.006) and 100 ng knee^−1^ (*P*=0.032) compared to control aptamers as measured by knee circumference 48 h after injection of aptamers and zymosan (Fig. 2b; *P* values determined by two-tailed, unpaired Student's *t*-test). Less pronounced but similar results were observed 24 h after injection (Supplementary Fig. 6). Histopathological assessment of DEK-targeted versus control aptamer-treated joints revealed a significant overall reduction in inflammatory cell migration as determined by pathological assessment of hematoxylin and eosin sections (Fig. 2c,d). Notably, fluorescent immunohistochemistry staining revealed fewer Ly6G-positive cells in DTA-64-treated knees (Supplementary Fig. 7A, B), similar to the phenotype of *Dek*-KO mice. In contrast, there was no significant difference in the infiltration of monocytes or macrophages as detected by the CD11b marker (Supplementary Fig. 7C). Screening of a panel of inflammatory cytokines showed significant reduction only in the levels of IL-1β and IL-6 when homogenates from DTA-64 and control aptamer treated knees were compared (Supplementary Fig. 7D). As IL-1 and IL-6 are both presently targets of clinically available therapeutics, the modest differences seen might yet be significant. Interestingly, NETs have recently been shown to promote IL-6 and IL-1β production by macrophages in the setting of atherosclerosis^19^. In summary, DEK-targeting aptamers can reduce neutrophil recruitment and the inflammatory response in the ZIA murine model.

### DEK is crucial for NET formation

**Figure 3:**
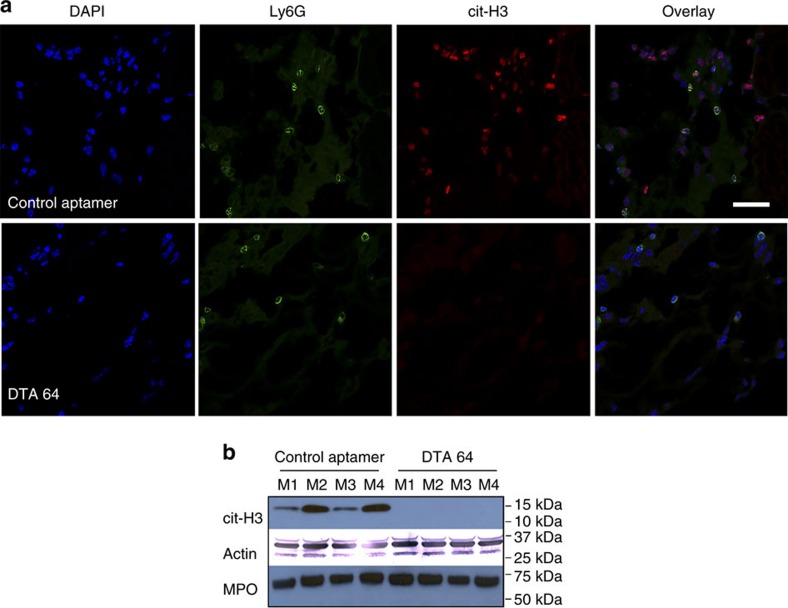
Anti-DEK aptamers reduce NET formation in zymosan-injected joints. (**a**) Representative confocal microscopy images of permeabilized joint sections from knees injected with zymosan and control aptamer (*n*=3) or anti-DEK aptamer (*n*=3) stained for Ly6-G (green) or the NET marker citrullinated histone H3 (cit–H3) antibody (red). Magnification × 60 (scale bar 20 μm). Staining for cit-H3 is undetectable in the neutrophils in the DTA 64-injected knees, but is readily seen in the control aptamer-injected joints. The absence of cit-H3 in the DTA-64 injected-knees correlates with the loss of NETs. (Please note that permeabilization of the cells makes it difficult to detect NET structures; please see NET staining in the non-permeabilized section with MPO in Supplementary Fig. 6E.) (**b**) Western blot analysis of knee joint homogenates from eight different mice (four joints/group) confirming dramatic decreases in cit-H3 in joints injected with DTA-64 as compared to control aptamer.

The reduction in neutrophil migration in the joints of mice injected with the anti-DEK aptamer as compared to those injected with the control aptamer was significant (Supplementary Fig. 7A,B), but not enough to explain the dramatic decrease in joint inflammation observed in the anti-DEK aptamer treated knees. Given the fact that DEK is found in the extracellular space and that DEK is important for chromatin architecture, we postulated that DEK may contribute to the functional integrity of extracellular chromatin structures termed NETs, which have been linked to the pathogenesis of autoimmunity^20^. Therefore, knee joint sections were co-stained for the neutrophil marker Ly6G and the NET marker citrullinated histone H3 (cit-H3) (cells other than neutrophils (possibly activated macrophages) also stained positive for cit-H3 (refs 21, 22)). Strikingly, no cit-H3 staining was detected in neutrophils found in the anti-DEK aptamer injected joints (Fig. 3a). In an attempt to visualize NETs in the joint tissues, we immunostained non-permeabilized sections of the knees from mice treated with DTA-64 or control aptamer with myeloperoxidase (MPO), also a known NET marker, revealing a significant reduction in extracellular MPO (Supplementary Fig. 7E,F). NET structures can be detected in some areas by DAPI and MPO, further suggesting that the reduction in MPO staining reflects a reduction in NETs in the DTA64-treated mice. Western blot analysis of joint homogenates showed no cit-H3 staining in the anti-DEK aptamer injected knees, in contrast to those injected with control aptamer (Fig. 3b and Supplementary Fig. 7G for full gel images). Intracellular levels of MPO were similar, probably due to similar recruitment of monocytes and macrophage as detected by CD11b staining (Supplementary Fig. 7C), as DEK does not affect monocyte or macrophage migration^6^.

**Figure 4:**
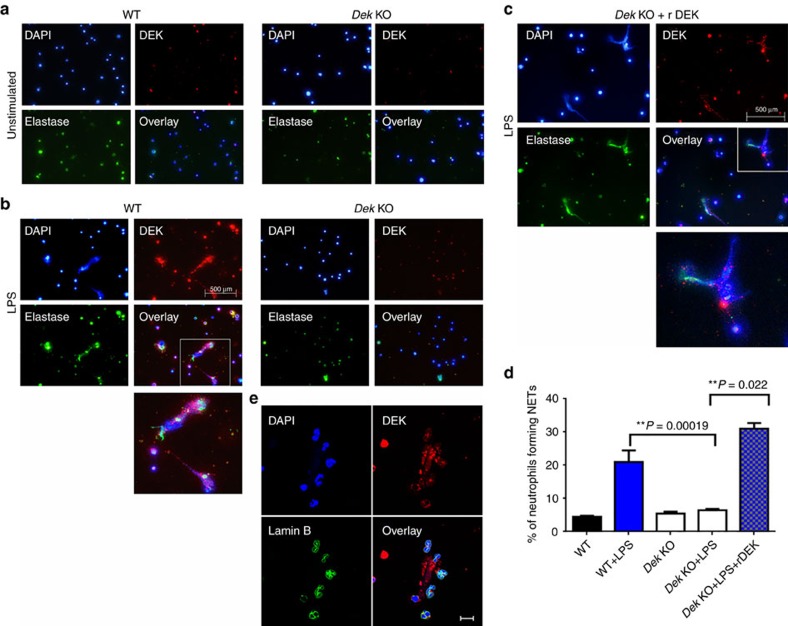
DEK is required for NET formation in murine neutrophils. Neutrophils were purified from the bone marrow of WT (*n*=3) or *Dek*-KO (*n*=3) mice and fixed and stained with DAPI (blue), rabbit anti-DEK (red) and mouse anti-neutrophil elastase (green). (**a**) Unstimulated neutrophils from BM WT and *Dek*-KO mice. (**b**) Neutrophils from WT mice stimulated with LPS for 2 h form NETs, whereas those from *Dek*-KO mice do not. (**c**) Addition of recombinant mouse DEK (3.5 μg ml^−1^) prior to LPS stimulation of *Dek*-KO neutrophils leads to the formation of NETs (× 40 magnification, scale bar 500 μm). (**d**) Percentage of neutrophils forming NETs per field in WT neutrophils as compared to neutrophils from *Dek*-KO mice with and without addition of recombinant DEK, as determined by two-tailed, unequal variance Student's *t*-test (error bars, s.e.m.). Results shown are representative of at least eight different fields counted for each condition, and represent three independent experiments counted by three different investigators. (**e**) Extracellular DEK does not enter the nucleus of neutrophils. BM neutrophils obtained from *Dek*-KO mice were incubated with recombinant DEK (3.5 μg ml^−1^) prior to PMA stimulation. Cells were fixed, permeabilized and stained with a monoclonal antibody to DEK (red), and the nuclear envelope was stained with an antibody directed against Lamin B (green). (× 60 magnification, scale bar 10 μm). Recombinant DEK is detected only on the cell surface and associated with NETs.

To further study the role of DEK in NET formation, we purified and stimulated neutrophils from the bone marrow of *Dek*-KO and WT mice to determine their capacity to generate NETs *in vitro*. Purity of bone marrow neutrophils from both WT and *Dek*-KO mice was confirmed by CD11b and Ly6G staining (Supplementary Fig. 2A,B). No obvious differences in neutrophil nuclear morphology or spontaneous NET formation were detected in unstimulated neutrophils from WT versus *Dek*-KO mice (Fig. 4a). However, neutrophils from *Dek*-KO mice showed only a very limited capacity to form NETs after LPS stimulation, as detected by extracellular co-localization of DAPI and anti-elastase antibody, when compared to neutrophils from WT mice (Fig. 4b,d; *P*=0.00019 as determined by two-tailed, unpaired Student's *t*-test). Interestingly, DEK staining was detected only in a few cells in the WT control neutrophils, but much more so after LPS stimulation. This difference can be explained by post-translational modification of DEK (as the monoclonal DEK antibody best recognizes phosphorylated DEK) or generally increased expression of DEK after stimulation. Importantly, loss of NET formation in the absence of DEK was noted even after phorbol myristate acetate (PMA) activation of neutrophils from *Dek*-KO and WT mice was extended for up to 8 h (Supplementary Fig. 8) and in peripheral blood neutrophils from *Dek*-KO mice (Supplementary Fig. 9). Furthermore, the observed defect in NET formation in the absence of DEK was not explained by a reactive oxygen species-driven mechanism, as no difference in H_2_O_2_ generation was noted between *Dek*-KO and WT mice (Supplementary Fig. 10A,B). Remarkably, however, reconstitution with recombinant DEK protein 1 h prior to activation with LPS rescued the ability of *Dek*-KO neutrophils to generate NETs (Fig. 4c,d).

**Figure 5:**
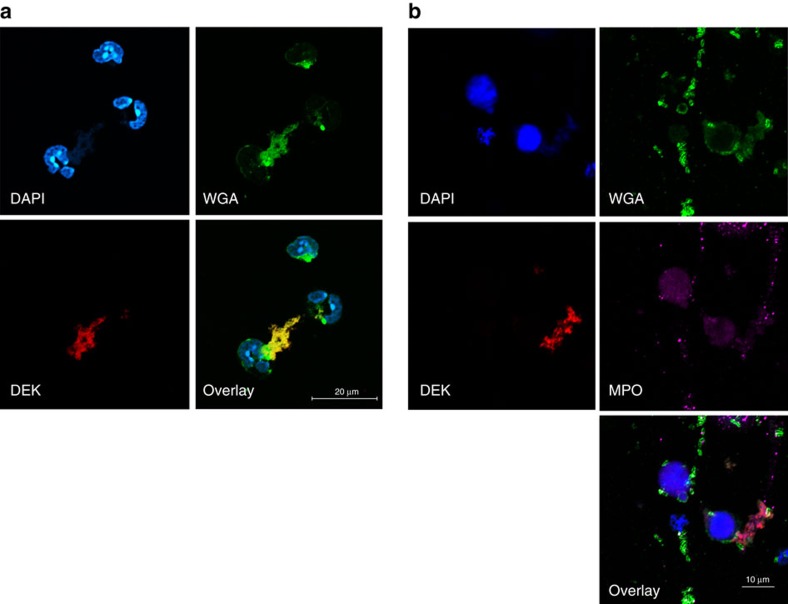
Extracellular recombinant DEK does not enter the cell but restores NET formation in *Dek*-KO neutrophils. *Dek*-KO (*n*=3) BM neutrophils were treated with recombinant DEK prior to PMA stimulation. (**a**) Cells were fixed 2-3 h after PMA treatment, and then permeabilized and stained for DEK (red) and wheat germ agglutinin (WGA), a membrane marker. DEK is detected outside of the cell in the NETs (× 60 magnification, scale bar 20 μm, Nikon Confocal). (**b**) Cells were also stained for the NET marker myeloperoxidase (MPO, purple), DEK (red) and WGA (green). DEK is detected outside of the cell in NETs (× 60 magnification, scale bar 10 μm, Nikon Confocal). Images are representative of three different fields from at least three independent experiments.

We have previously demonstrated that DEK is crucial to global heterochromatin integrity within the nucleus^7^. Since NETs are chromatin-containing structures, DEK could affect chromatin structure and hence NET formation by one of two basic mechanisms: (1) DEK is known to modulate intranuclear chromatin structure, and in a number of different cell types recombinant DEK is taken up by cells and can go directly to the nucleus and affect chromatin structure and cell function^7^,^8^. In this capacity, DEK could participate in the early events of NET formation. (2) DEK could affect NET formation in the cytoplasm or extracellular space by serving as a scaffold. To test if extracellular DEK enters the nucleus of *Dek*-KO neutrophils to restore NET formation, we added recombinant DEK and stained the nuclear envelope with Lamin B. As shown in Fig. 4e, recombinant DEK does not enter the nucleus of the neutrophil, but simply associates with the NET structures. In addition, we stained the cells with wheat germ agglutinin, a plasma membrane marker, MPO and anti-DEK antibody, and saw that, again, recombinant DEK added to neutrophils not only did not enter the nucleus, but actually did not enter the cell and was found in the extracellular space (Fig. 5a,b). Thus, DEK does not appear to promote NET formation from within the cell, but rather acts as a key component of NET architecture in the extracellular space. Denaturation of recombinant DEK protein prior to addition to *Dek*-KO neutrophils prevented proper restoration of NET formation (Supplementary Fig. 11). Taken together, these findings suggest that bioactive DEK plays an important role in NET formation, likely through its effects on the extracellular chromatin component that frames these structures.

### DEK is detected in human NETs

**Figure 6:**
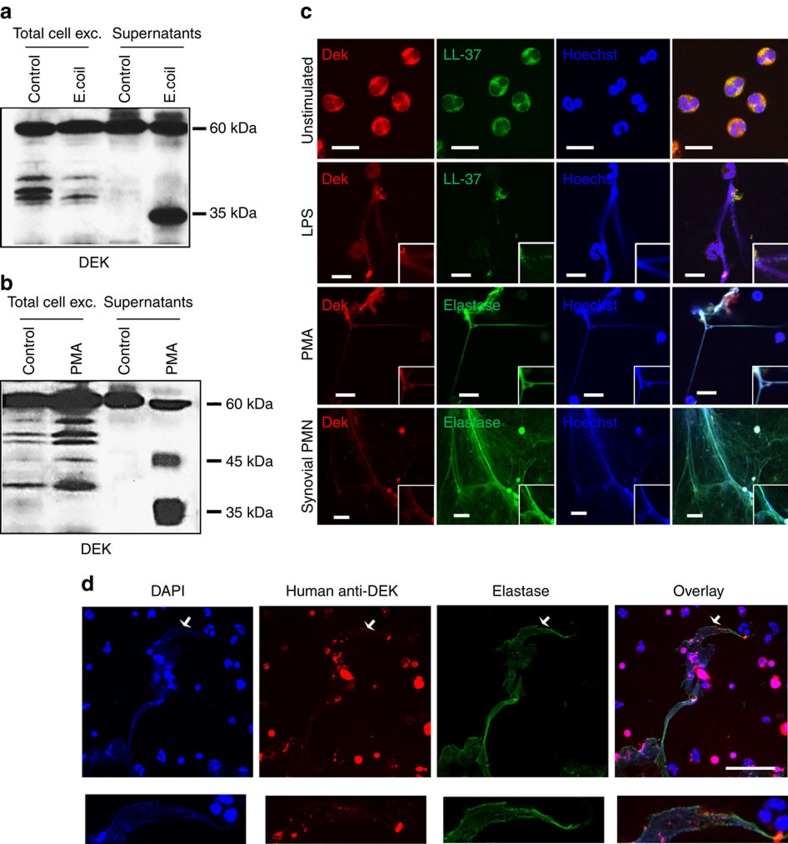
DEK is released into the extracellular space by human neutrophils and is found in NETs. 1 × 10^7^ human neutrophils (from two different healthy individuals) in serum-free media were left unstimulated or were stimulated with *E. coli* (**a**) or PMA (**b**). Supernatants and cells were harvested after 2 h of incubation and were analysed by immunoblotting using a rabbit polyclonal antibody specific for DEK. DEK is detected as a 45 kDa and/or a 35 kDa protein, the latter of which is a well-known naturally occurring breakdown product of DEK. The 60 kDa form of DEK is most likely the result of posttranslational modifications (see discussion in text). Results are representative of neutrophils from at least three different healthy human volunteers. (**c**) Neutrophils were isolated from the peripheral blood of healthy volunteers (upper three panels) or from the synovial fluid of a JIA patient (lower panel). Unstimulated neutrophils, or LPS or PMA-stimulated neutrophils, were stained with Hoechst (blue) for DNA, mouse or rabbit antibody to DEK (red), rabbit anti-neutrophil elastase (green) or mouse anti-LL-37 antibody (magnification × 63, scale bar 10 μm). The results of the experiments shown are representative of those seen with the neutrophils from the synovial fluids of three patients with JIA. (**d**) DEK autoantibodies purified from the synovial fluid of a JIA patient recognize DEK (red) in spontaneously formed NETs from synovial fluid neutrophils. DNA is stained with DAPI (blue) and the NET-specific marker elastase is detected by antibody and stained green (magnification × 60, scale bar 50 μm). All pictures were taken by confocal microscopy (Nikon Confocal microscope). The lower panel zooms in on the NETs marked by the arrows in the upper panel.

Our *in vivo* studies in mice led us to next investigate the relevance of our findings to human biology. We first examined the possibility that activated human neutrophils release DEK into the extracellular space. Indeed, stimulation of primary human neutrophils from healthy donors with *Escherichia coli* (*E.coli*) (Fig. 6a) or 10 ng ml^−1^ PMA (Fig. 6b) (see Supplementary Fig. 12 for full gel images) led to the release of DEK into the extracellular milieu. The banding patterns demonstrated by immunoblot analysis are consistent with previously reported findings of numerous DEK isoforms in primary cells^6^,^23^. Exposure of fresh neutrophils to *E.coli* or PMA for 2 h primarily induced the release of the 35 and 45 kDa forms of DEK, suggesting that DEK is modified as a result of neutrophil activation by *E.coli* or PMA. A 60 kDa form of DEK is always detected in the supernatant and in cell extracts of the unstimulated cells. To understand if DEK released into the extracellular space is associated with NETs, human peripheral blood neutrophils were subjected to LPS or PMA treatment to induce NET formation; immunohistochemistry revealed co-localization of DEK with the known NET markers LL-37 and neutrophil elastase^24^ (Fig. 6c).

**Figure 7:**
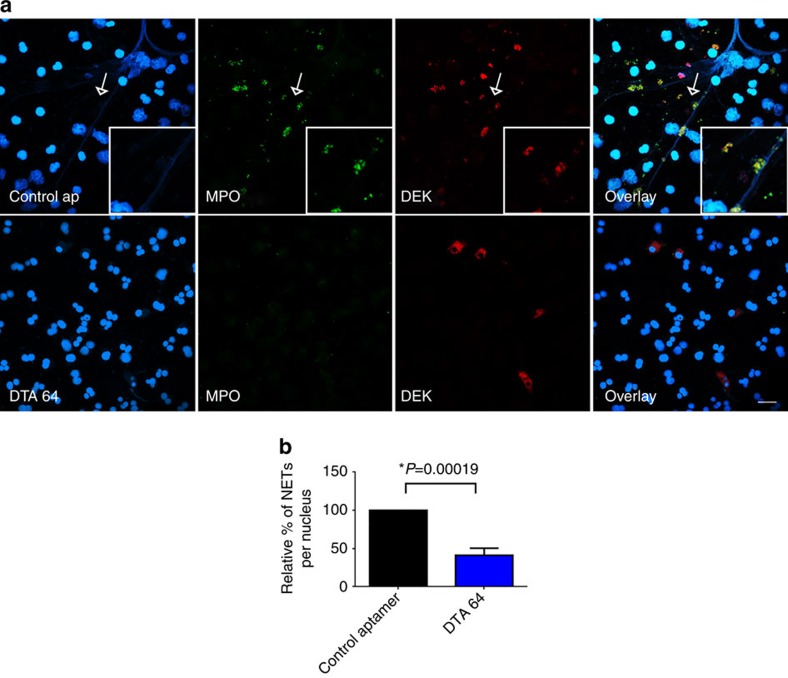
Neutrophils treated with anti-DEK aptamer (DTA-64) have fewer NET structures and show retention of intracellular DEK. (**a**) Peripheral blood human neutrophils were obtained from healthy donors and plated on glass coverslips. Control or anti-DEK aptamers (DTA-64) were added to neutrophil cultures 15 min prior to PMA stimulation (10 ng ml^−1^). Neutrophils were incubated for 1 h at 37 °C, then fixed and stained for NETs with anti-MPO (green), DAPI for DNA (blue) and DEK (red) as shown in representative confocal images from experiments using 5 ng ml^−1^ of control aptamer or DTA-64 (× 60 magnification, scale bar 20 μm). The inserts zoom in on the NETs marked by the arrows in the upper panel. (**b**) NET staining intensity based on MPO and DAPI was determined by Metamorph 7.7. Results shown represent the average from the neutrophils of five different healthy donors. The number of NETs/nucleus is markedly reduced (*P=0.00019) in neutrophils treated with anti-DEK aptamer as compared to control aptamer as determined by two-tailed, unequal variance Student's *t*-test (error bars, s.e.m.).

**Figure 8:**
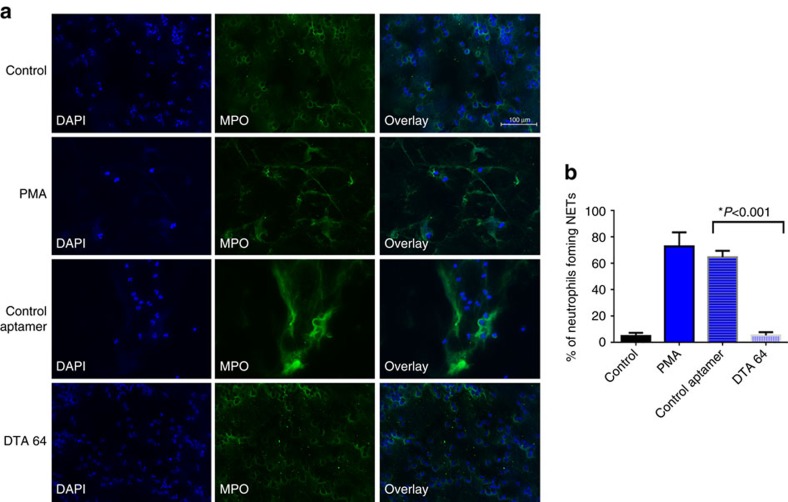
DEK aptamers block NET formation by activated human neutrophils after 4 h of stimulation with PMA. (**a**) Peripheral blood human neutrophils were obtained from healthy individuals and plated on glass coverslips. Aptamers to DEK were added at 50 ng to the neutrophil culture prior to PMA stimulation (10 ng ml^−1^). Neutrophils were incubated for 4 h at 37 °C, then fixed and stained for NETs with anti-MPO antibodies (green) and DAPI for DNA (blue) (× 20 magnification, scale bar 100 μm). (**b**) Percentage of neutrophils forming NETs per field following treatment with control library aptamer as compared to neutrophils treated with anti-DEK aptamer (DTA 64) (*P<0.001 as determined by two-tailed, unequal variance Student's *t*-test ) (error bars, s.e.m.). Results shown are based on three different experiments from three different healthy donors and a total of 13 different fields counted by two different investigators.

To investigate whether DEK is present in NETs released into the joints of JIA patients, we purified neutrophils from SFs and immediately subjected them to immunocytochemical analysis using monoclonal anti-DEK antibody (Fig. 6c, bottom). We observed that neutrophils purified from SFs of JIA patients are already significantly activated, such that they form NETs without further stimulation. The NETs showed positive DEK staining, and significant co-localization of DEK with elastase, LL-37 and DNA (stained by Hoechst). Further, affinity-purified DEK autoantibodies isolated from the SFs of JIA patients recognized NETs formed by synovial neutrophils (Fig. 6d); we have previously demonstrated that these autoantibodies show specific recognition of DEK^5^. Finally, incubation with the anti-DEK aptamer DTA-64, but not control aptamer, blocked formation of PMA-induced NETs by human peripheral blood neutrophils from healthy control individuals as detected by MPO staining and DAPI. DEK is detected mainly in the extracellular space and associated with NETs in the control aptamer-treated cells, but after DTA-64 treatment it is detected primarily in the cytoplasm of neutrophils (Fig. 7a). NET formation in neutrophils treated with DTA-64 aptamer prior to PMA stimulation was significantly inhibited, by 60% as compared to control aptamer (*P*<0.0001 as determined by two-tailed, unpaired Student's *t*-test), using neutrophils from five different donors (Fig. 7b). Dose-response experiments show that the effect of anti-DEK aptamer can generally be detected at 1 ng to 50 ng ml^−1^ after 1 h of PMA treatment (Supplementary Fig. 13A,B) and 4 h after PMA treatment (Fig. 8a,b). NET formation was evaluated by manual counts and computer analysis as described in the Methods section. (The SYTOX Green assay was not used here as it was found to also stain the aptamers, as they are extracellular small DNA sequences.) These results further support the important role of DEK in NET formation and inflammation in humans, and suggest that it is a target of interest in the treatment of inflammatory diseases.

## Discussion

DEK is an autoantigen in JIA and other autoimmune diseases^12^,^23^,^25^, and is found at high levels in the joints of children with JIA, often complexed with anti-DEK antibodies^5^. Previous work had shown a potentially direct role for DEK in inflammation, as this biochemically distinct protein, usually found in the nucleus, can be secreted by macrophages and released by apoptotic T cells and then function as a chemoattractant for neutrophils and other inflammatory cells^6^,^26^.

Our genetic studies now reveal that DEK is crucial for the development of inflammation *in vivo*. We have used a mouse knockout model to demonstrate directly that DEK facilitates development of inflammatory arthritis *in vivo.* Using the ZIA mouse model^17^, we found that *Dek*-KO mice are significantly less likely than WT mice to develop arthritis, and single-stranded anti-DEK DNA aptamers markedly attenuate inflammation in WT mice subjected to ZIA. Consistent with this observation, we also found that homogenates of zymosan-injected joints from *Dek*-KO versus WT mice had significantly lower levels of inflammatory cytokines such as IL-1α, TNF-α and RANTES, the latter of which can be produced by T cells in response to TNF-α and IL-1α. These results further support the significant differences in the inflammatory reaction observed in the respective animals (Fig. 1a). It is of particular note that IL-1α and TNF-α, inflammatory cytokines that have been targeted very successfully in the treatment of JIA, RA and other autoimmune diseases^27^,^28^, are produced in greater abundance in WT as compared to *Dek*-KO mice. The lower levels of IL-12p40, IL-12p70 and IL-23 in the joints of *Dek*-KO versus WT mice injected with zymosan also suggest that DEK has an effect on T-cell response.

We have now found that, like macrophages and T cells, neutrophils can release DEK into the extracellular space. Our initial idea was that DEK would induce inflammation *in vivo* by recruiting white blood cells. This is only partially correct. The total white blood cell, including neutrophil, recruitment was significantly reduced after treatment with anti-DEK aptamers, but only a modest though significant decrease was observed in neutrophil chemotaxis in the ZIA model when WT and *Dek*-KO mice were compared (Fig. 1b,c). This suggests that other functions of DEK contribute to the marked reduction in inflammation seen when DEK is functionally inactivated by genetic depletion or aptamers. As DEK is also crucial to chromatin biology within the cell^7^, we asked whether DEK might take part in NET formation. Strikingly, we found that DEK is vital to these extracellular structures that regulate innate immunity and the generation of autoantibodies. Using specific polyclonal DEK antibodies and DEK autoantibodies from JIA patients, we detected DEK in human NETs. NETs were spontaneously formed by JIA synovial neutrophils, likely due to their activated state in these patients with autoimmune disease.

The majority of proteins that are found in NETs originate from granules, and a few are nuclear/chromatin proteins, such as HMGB1 (ref. 24). Given the importance of chromatin to the composition of NETs, the role of DEK in chromatin biology^7^, and the presence of DEK in the NETs from activated neutrophils, it was imperative to investigate the importance of DEK to the genesis of NETs. We found that neutrophils from *Dek*-KO mice almost completely failed to form NETs after short-term and long-term (up to 8 h) stimulation with PMA. Unexpectedly, simple addition of recombinant DEK allowed *Dek*-KO neutrophils to create fully formed NETs without entry of DEK into the cytoplasm or the nucleus. Further, treatment of activated human neutrophils with anti-DEK aptamer resulted in a marked loss of their ability to generate NETs. These striking results indicate that DEK plays a vital role in the formation of NETs. Moreover, *Dek*-KO neutrophils mature, differentiate and express reactive oxygen species activity, as well as MPO and elastase proteins, similarly to WT neutrophils. Therefore, it appears that the key role of DEK in NETosis is as a chromatin architectural factor. We have previously demonstrated that DEK is crucial to global chromatin integrity within the nucleus^7^, and in the present study we show that it is vital to NETs, which are extracellular chromatin-containing structures. Thus, the pro-inflammatory DEK protein plays a role in the biology of both intracellular and extracellular chromatin. NETs have been proposed to act as a double-edged sword^20^ in innate immune responses: they are microbicidal and may help to control infection following exposure to microorganisms, yet enhanced NETosis has been found to contribute to the development of various autoimmune diseases, including SLE (ref. 29). Further, NETs are involved in increased cytokine production, which could explain our findings that DEK regulates the production of both inflammatory cytokines and of NETs (refs 19, 30, 31, 32).

Aptamers are an exciting approach to the treatment of diseases in which specific molecular targets can be identified. Presently, an anti-vascular endothelial growth factor aptamer is FDA-approved for use in the treatment of macular degeneration^3^,^4^. There are also aptamers under development that attack pro-inflammatory molecules, such as neutrophil elastase and NF-κB. Several aptamers have also reached Phase I and II clinical trials for the treatment of acute myeloid leukemia, thrombosis (thrombotic microangiopathies), carotid artery disease, multiple-myeloma, non-Hodgkin's lymphoma, type 2 diabetes and diabetic nephropathy^33^. The ongoing development of clinically useful aptamers and the present study support the idea that targeting DEK with aptamer therapy is a potentially useful approach to treating autoimmune diseases once optimization is accomplished. JIA is particularly appealing as the pathogenesis of this constellation of diseases is poorly understood and so specific therapeutic targets are lacking. The genetic and aptamer experiments presented in this report clearly identify DEK, a protein with a complex and unusual intracellular/extracellular life-cycle, as a key mediator of inflammation.

## Methods

### Mice

*Dek* knockout (KO) mice (129/SVEV on C57BL/6) were kindly provided to us by Dr Gerard Grosveld from St Jude Children's Research Hospital. Control mice were generated by back-breeding *Dek*-KO mice with the C57BL/6 WT strain for a minimum of ten generations, bred primarily as heterozygotes. Mice were housed in specific pathogen-free conditions at the Animal Maintenance Facility of the University of Michigan Medical Center, until they were used for experiments at 10–13 weeks of age with a minimum of five mice allocated in a randomized and blinded manner, per group per experiment. Random male and female mice were used in all the experiments. The University of Michigan Committee on Use and Care of Animals reviewed and approved all animal protocols (PRO00005124).

### Zymosan-induced arthritis (ZIA)

Zymosan A from *Saccharomyces cerevisiae* (Sigma, St Louis, MO, USA) (30 mg) was resuspended in 2 ml of endotoxin-free saline, and was subsequently boiled and homogenized by sonic emulsification. Arthritis was induced by intra-articular (i.a.) injection of 300 μg (20 μl) of zymosan through the suprapatellar ligament into the joint space. In specified experiments, the contralateral knee was injected with an equal volume of sterile saline (20 μl) as a control. Thirteen-week-old female and male mice WT (11 females and 13 males) and *Dek*-KO (12 females and 15 males) were injected i.a. with zymosan into the knees of both hind legs on day 0. Knee circumference was measured by two different investigators in a blinded fashion before injection on day 0, and at 24 and 48 h after injection. The degree of arthritis was indicated by joint swelling and quantified by knee circumference, determined by measuring two perpendicular diameters of the joint with calipers (Lange Caliper, Cambridge Scientific Industries, Cambridge, MA, USA). Knee circumference was determined in a blinded fashion using the following geometric formula: circumference=2π (✓(*a*^2^+*b*^2^/2)), where *a* is the latero-lateral diameter, and *b* is the antero-posterior diameter^34^.

### Knee homogenates

Knees designated for homogenization were skinned prior to freezing at −80 °C. Frozen knees from WT (5 females and 2 males) and *Dek*-KO (7 females and 4 males) were homogenized in 0.5 ml of cold phosphate-buffered saline (PBS) and then centrifuged at 14,000*g* for 10 min at 4 °C. Supernatants were collected and analysed for protein concentration by Western blot analysis and ELISA for levels of mouse IL-1α, IL-1β, TNF-α, IL-12p70, IL-12 p40, IL-23, RANTES (Regulated on Activation Normal T cell Expressed and Secreted), MIP-2, IL-10, MCP-1, IFNγ and TGFβ (ref. 35).

### DEK targeting aptamers

We generated anti-DEK aptamers by the SELEX technology, a method that selects for either single-stranded DNAs or RNAs that bind tightly to the protein of interest and can potentially inactivate its function^36^,^37^. SELEX is performed using multiple rounds of selection and involves screening vast numbers of random DNA or RNA sequences to find the sequence of interest (see below for a complete description). In doing so, we identified a 41 nucleotide, single-stranded DNA—5′-GGG GTT AAA TAT TCC CAC ATT GCC TGC GCC AGT ACA AAT AG-3′—DEK targeting aptamer 64 (DTA-64), with high affinity for recombinant DEK protein (produced in a baculovirus system^38^), which binds very tightly to DEK (Supplementary Fig. 5). Aptamers to DEK or a control from the library (scramble/random sequence) were diluted in PBS to a concentration of 5–500 ng per 20 μl volume for i.a. injection. Fifty-six WT mice (22 males and 34 females), 12-13-week-old mice (129/SVEV on B6) obtained from Jackson laboratory at age of 10 weeks were injected with aptamers 30-60 min before zymosan injection. Circumferences of the knees were measured 24 and 48 h after injection as described above. Mice were euthanized 48 h after injection, and knees were harvested for histology and pathological assessment. Investigators were blinded to group allocation during the experiment and when assessing the outcomes. Knee homogenates (described above) were also analysed by western blot (24 μg per lane), with the polyclonal antibody cit-H3 (1:1,000) and monoclonal β-actin antibody used as loading controls.

### DEK aptamer screen and selection

A pool of 86-nucleotide DNA oligomers containing 40 central nucleotides of random sequence flanked by defined primer-binding sites (Supplementary Fig. 5) was synthesized to order by Integrated DNA Technologies (Coralville, IA, USA). This resulted in an initial pool with estimated complexity of 10^14^–10^16^ different sequences: (5′-ATAGGAGTC- GACCGACCAGAA [N]40 TATGTGCGTCTACATCTA- GACTCAT-3′) (SEQ ID NO: 3). Short DNA oligonucleotides for amplifying selected sequences were: 5′-primer, 5′-ATAGGAGTCGACCGACCAGA A (SEQ ID NO: 4); 3′-primer, 5′-ATGAGTCTAGATGTAGACGCACATA (SEQ ID NO: 5).

Round 1: For the first round of selection, approximately 1 mg of randomized single-stranded DNA library (Integrated DNA Technologies, Corralville, IA, USA) was incubated with 1.5 ml bed volume of nickel-nitrilotriacetic acid (Ni-NTA) agarose resin (Qiagen, Germantown, MD, USA) conjugated to histidine-tagged DEK protein (at ∼1 mg DEK per ml of resin) in 5 ml of binding buffer (20 mM Tris pH 7.6; 100 mM NaCl; 5 mM MgCl_2_). The library was allowed to bind to the resin-conjugated DEK for 1 h at room temperature on a rotating wheel. The resin was spun down gently in a swinging-bucket centrifuge and the supernatant was removed. In fresh binding buffer, the resin was transferred to a clean 50-ml conical tube and washed four times with 25 ml binding buffer, 15 min each wash, transferring to a clean conical tube after the second wash. To elute, the resin was transferred to a clean 15 ml conical tube and incubated with 1.5 ml elution buffer (20 mM Tris pH 7.6; 5 mM MgCl_2_; 1 M NaCl; 7 M urea) for 1 h on a rotating wheel at room temperature. The resin slurry in elution buffer was applied to 0.45 μm spin columns (Millipore, Damrstadt, Germany), and spun in a microcentrifuge to separate the eluate from the beads. An additional 500 μl of elution buffer was applied to the 15-ml conical tube to recover any additional beads that adhered to the sides during the elution step; this too was spun through the spin columns. Thus, the total recovery volume was ∼2 ml. This volume was then extracted twice, with equal volume phenol:chloroform:isoamyl alcohol (25:24:1), extracted once with equal volume chloroform, then precipitated with 2.5 volumes of ethanol and resuspended in 20 mM Tris pH 7.6.

To amplify aptamers that bind to DEK, asymmetric PCR amplification was performed with DNA oligonucleotide primers complementary to end sequences (5:1 ratio of 5′ primer to 3′ primer) (5′-primer, 5′-ATAGGAGTCGACCGACCAGA A (SEQ ID NO. 4); 3′-primer, 5′-ATGAGTCTAGATGTAGACGCACATA; Integrated DNA Technologies, Coralville, IA, USA). The amplified DNA pool was then gel purified through denaturing polyacrylamide gel electrophoresis (PAGE) and soaking elution/ethanol precipitation to proceed to the next round.

Round 2: One-fourth of the resin from the Round 1 step (approx. 375 μl bed volume) was used for Round 2 and subsequent rounds. The resin was washed in binding buffer and transferred to a pre-lubricated Eppendorf tube (Sorenson BioScience, #11700, Salt Lake City, UT, USA). To this resin was added the amplified and gel-purified DNA pool from Round 1. This mixture was allowed to bind for 1 h at room temperature on a rotating wheel. The mixture was then transferred to a 0.45 μm spin column and the unbound fraction was spun out. The resin was washed four times, each in 500 μl binding buffer, keeping the resin in the spin column during the washes, inverting vigorously several times each, and spinning out. To elute, with the resin still in the column, 200 μl of elution buffer was added, and eluted for 15 min on a room temperature rotating wheel. The eluate was spun through in a microcentrifuge to separate away from the resin. In order to dilute the salt and the urea, 200 μl of water was added to the eluate prior to proceeding with ethanol precipitation.

Asymmetric PCR amplification was performed with DNA oligonucleotide primers complementary to end sequences (5:1 ratio of 5′ primer to 3′ primer) as above. The amplified DNA pool was then gel purified through denaturing PAGE and soaking elution/ethanol precipitation before proceeding to the next round.

Rounds 3-5: Prior to proceeding to Round 3, a subtraction was performed to remove nonspecific binders. One hundred microlitres of Ni-NTA slurry (with no DEK conjugated) was washed once in binding buffer and added to the Round 2 DNA pool in fresh binding buffer. This mixture was incubated for 30 min on a rotating wheel at room temperature to allow nonspecific binders to separate. To continue with Round 3, the supernatant was spun out and added to the same DEK-conjugated resin used for Round 2. Round 3 proceeded exactly as Round 2.

After PCR and gel purification, Round 4 was performed without subtraction. Between Rounds 4 and 5, a second subtraction step was performed as above. Round 5 selection was done as with other rounds. Asymmetric PCR amplification was performed with DNA oligonucleotide primers complementary to end sequences (5:1 ratio of 5′ primer to 3′ primer) as above. The amplified DNA pool was then gel purified through denaturing PAGE and soaking elution/ethanol precipitation to proceed to the next round.

Round 6: The gel-purified DNA pool from Round 5 was radiolabelled to provide visualization via native PAGE gel shift. Approximately 1 million counts (<1 μg) of labelled DNA was incubated with titrated amounts of soluble (that is, not conjugated to [his x6]) DEK protein (starting at 128 ng, with two-fold dilutions down to 1 ng) in 10 microlitres. Bound DEK-DNA complex was separated from free DNA on a 6% native gel supplemented with 5 mM MgCl_2_ and 5% glycerol. From the lane that gave the greatest percentage shift of DNA in a single band, the bound complex was excised and eluted in 20 mM Tris pH 7.6.

The Round 6 DEK pool was amplified via asymmetric PCR (5:1 (250 μM:50 μM) ratio of 5′ primer to 3′ primer) and radiolabelled following the T4-polynucleotide kinase (PNK) protocol (New England Bioscience, Ipswich, MA, USA): 1 μl T4 PNK, 1 μl ^32^P ATP (3,000 Ci mmol^−1^, 5 mCi ml^−1^), 2 μl 10 × T4 PNK buffer, 1 μg DNA, and H_2_O up to 20 μl). The radiolabelled DNA was separated from the unincorporated ^32^P via a 6% denaturing gel. The bands corresponding to the correct size were excised and soaked in 400 μl of water overnight. The DNA was then precipitated and re-suspended in water. The amount of ^32^P labelled DNA in each tube was determined using a scintillation counter (Beckman LS6500, Beckman Coulter, Indianapolis, IN, USA).

To test binding of the radiolabelled pools (DEK round 6) to the DEK protein, a dot blot was performed. Glass fibre filter paper A (Whatman/GE Healthcare, Pittsburgh, PA, USA) and DEAE filters (Whatman/GE Healthcare) were soaked in binding buffer for 1 h prior to use. One hundred nanograms of DEK protein was incubated in binding buffer and 100 μg ml^−1^ of salmon sperm DNA for 15 min. Five thousand CPM of the radiolabelled DNA pool was incubated with the DEK and salmon sperm solution on a rotator for 1 h. The dot blot vacuum filter apparatus was set up with the DEAE paper on the bottom and the GFC/A paper on top. Each well was washed with 100 μl of cold binding buffer (see Round 1 above). One hundred microlitres of the radiolabelled pool/DEK protein solution was placed into each well eight samples at a time, and immediately vacuumed until the solution passed through the membrane. The wells were then immediately washed with 100 μl of cold binding buffer. When all of the samples were loaded, all of the wells were washed 3 × with 200 μl of binding buffer. The membrane was then dried, and signal determined using a Typhoon phosporimager (GE Healthcare, Pittsburgh, PA, USA) for 1–2 h to determine individual aptamers that had signal above background of the same reaction without DEK protein.

To obtain ‘monoclonal' aptamers with individual sequences and properties: Cloning was performed following the protocol in the Promega pGEM-T kit (Model A3600, Madison, WI, USA) using a 3:1 molar ratio of final aptamer pool (made double-stranded by PCR) to pGEM plasmid, and the electroporation transformation (BioRad Gene Pulser), according to the manufacturer's instructions (Hercules, CA, USA). After plating on LB/Amp media and incubating overnight, white colonies were randomly selected and placed into a symmetric 1:1 (125 μM:125 nM) 5′ primer to 3′ primer PCR reaction. (Symmetric PCR conditions: 94 °C 5′ [94 °C 30 s, 55 °C 30 s, 72 °C 30 s] × 20 rounds, 72 °C C 10′, 4 °C hold) using the 5′ and 3′ primers as above.

After the symmetric PCR was complete to create double-stranded DNA corresponding to the DEK-binding aptamers, the DNA was gel-isolated through 6% denaturing polyacrylamide gels, soaked out into water, and ethanol precipitated with 2.5 volumes of ethanol. This DNA was used with an approximately 5 × molar excess of only the 5′ primer (sequence same as above) that had been radiolabelled to approximately 10,000,000 counts per microgram labelling with T4 polynucleotide kinase and gamma-^32^P-ATP as above. The asymmetric PCR (repeated unidirectional primer extension) components were as described in Supplementary Table 1. The asymmetric PCR conditions were: 94 °C 5′′, [94 °C 30′′, 59 °C 0′′, 72 °C 30′′] × 15 rounds, 72 °C 7′′, 4 °C hold.

ssDNA aptamers were amplified from the 96 colonies with single-stranded asymmetric PCR products radiolabelled as above and tested to determine their binding ability to DEK. Dot blots were performed as in DEK Round 6 binding above. Dot blots of aptamers giving the highest binding signal were repeated in quadruplicate to verify reproducibility.

The corresponding DNA of high affinity aptamer positive clones was purified using QIAprep spin miniprep kits (Qiagen) and sequenced by the University of Michigan DNA Sequencing Core. Ultimately, the final product used in the experiments shown was a 41 base anti-DEK aptamer, without the flanking sequences, which contains the following core sequence: 5′ GGG GTT AAA TAT TCC CAC ATT GCC TGC GCC AGT ACA AAT AG 3′. This aptamer was termed DTA-64. 

### Purification of human neutrophils

Forty millilitres of venous blood were collected from eight different healthy volunteer (four females and four males age 25–30) by consent as approved by the Institutional Review Board (IRB) of the University of Michigan (HUM00048623), into a 60 ml sterile syringe containing 7 ml 0.25 M Citrate (0.17 M sodium citrate and 0.083 M citric acid) and 6% Dextran in PBS buffer (without calcium or magnesium). The blood was incubated for 30 min at room temperature prior to collecting the upper phase by Histopaque-1077 (Sigma, St Louis, MO, USA) and centrifugation for 30 min at 700*g*. The neutrophil fraction was resuspended in 10 ml HBSS buffer, after which it was layered on Histopaque-1119 (Sigma, St Louis, MO, USA) for an additional 30 min separation by centrifugation at 700*g*. The neutrophil fraction was collected, washed once in HBSS and resuspended to a concentration of 500,000 cells ml^−1^ coverslip^−1^ in RPMI supplemented with 2% BSA. Cells were mounted on 22 × 22 mm, 2.5 μm glass coverslips treated with 0.001% poly-L-Lysine (Sigma, St Louis, MO, USA). A 1-h treatment with LPS (1 μg ml^−1^) or PMA (10 ng ml^−1^) (Sigma, St Louis, MO, USA) was used to induce NET formation. Cells were fixed in 4% paraformaldehyde/PBS, pH=7, prior to immunohistochemical examination. For immunoblot analysis, cells were lysed in 2% SDS buffer and supernatants were collected and concentrated using ultrafiltration spin columns with a 10 K molecular weight cut off^6^. Membranes were subjected to western blot analysis and probed with a DEK-specific polyclonal antibody generated in a rabbit (custom made) at a 1:1,000 dilution^5^.

### Purification of mouse neutrophils and monocytes

Harvested bone marrow from WT mice (8 females and 10 males) and *Dek*-KO mice (10 females and 8 males) was rinsed with 50 ml PBS prior to centrifugation at 500*g* × 5 min. The pellet was re-suspended in 5 ml PBS and cells were layered on a discontinuous gradient of 1 ml Histopaque-1119 and 5 ml Histopaque-1083. Tubes were centrifuged at room temperature (without brake) at 700*g* × 30 min. The top layer containing the monocytes was removed and the final 1.5–2 ml containing the neutrophils was rinsed with 50 ml PBS prior to repeat centrifugation at 500*g* × 5 min. Cells were re-suspended in 5 ml PBS prior to being counted and prepared for immunohistochemistry as described above and below, with the exception that rather than a 1 h incubation as for human neutrophils, the mouse neutrophils were incubated for 2 h with 1 μg ml^−1^ LPS to induce NETs. Monocytes were cultured at 0.2 × 10^6^ cells per ml and plated in six-well non-tissue culture plates in RPMI medium supplemented with 20% heat-inactivated fetal bovine serum (FBS) and 30% L-cell supernatant (a mouse fibroblast cell line that secretes M-CSF). Macrophages were harvested after 5–7 days of culture.

### Immunohistochemistry

Frozen sections of mouse joints were thawed rapidly and then fixed in 2% paraformaldehyde/PBS (pH=7.4) for 12 min at room temperature. Sections were washed twice for 2 min in PBS. Sections were washed with PBS three times for 5 min each, followed by blocking with 10% normal goat serum overnight at 4 °C. Sections were probed with rabbit anti-MPO (Dako Denmark A0398) at a dilution of 1:500, rat anti-Ly6C/Ly6G antibody (BD Pharmingen 550327) at a dilution of 1:50 overnight at room temperature, rabbit anti-cit-H3 (Abcam Inc., ab5103) at a dilution of 1:500, monoclonal anti-DEK (BD Pharmigen 610948) 1:50, or with custom-made rabbit anti-DEK antibody^6^ diluted in 10% normal goat serum at a dilutions of 1:50, 1:100 or 1:200 overnight at 4 °C. After washing in PBS for 5 min × 3, sections were incubated with secondary antibody; goat anti-rat IgG-AlexaFluor 594 (Invitrogen A-11007), at 1:200 for Ly6C/Ly6G or goat anti-rabbit IgG-AlexaFluor 594 (Invitrogen A-11037) at 1:200 for DEK and anti-cit-H3. Antibodies were diluted in 10% normal goat serum; sections were incubated for 45 min at room temperature.

### Identification and quantification of NETs

Neutrophils obtained from the peripheral blood of healthy volunteers, from SFs of JIA patients, or from mice were placed on coverslips as described above. Cells were stained with rabbit anti-DEK antibody (1:100) or mouse anti-DEK antibody (1:50 or 1:500, BD Bioscience, 610948), mouse monoclonal anti-elastase (1:500, Abcam, Cambridge, MA, USA ab78187), rabbit anti-elastase (1:1,000, Abcam, ab 21595), mouse anti-LL-37 (1:100, Abcam, ab64892), or rabbit anti-MPO (1:500, Dako A0398) at room temperature for 1 h followed by incubation with AlexaFluor 488 goat anti-rabbit or AlexaFluor 594 goat anti-mouse antibody (Invitrogen). Nuclei and NETs were visualized by DAPI—Prolong gold antifade (Invitrogen P-36931) or stained with Hoechst. Slides were analysed using a fluorescence microscope (BX; Olympus) or confocal microscope (Nikon) including Z-stacks of 80 0.3 micron optical sections (× 60). Ten high-power (× 40) images were captured. Images were loaded onto Adobe Photoshop (Adobe System), and NETs were counted manually and shown as a percentage of total neutrophils per field. NETs were counted by at least three independent observers in a blinded fashion and identified based on overlap of DAPI staining with the NET markers elastase and MPO.

### Purification of neutrophils from JIA synovial fluids

SFs were obtained from three different paediatric JIA patients (de-identified samples) during therapeutic arthrocentesis by trained medical staff of the Pediatric Rheumatology division at the University of Michigan. SFs were diluted 1:1 with PBS followed by separation on Histopaque-1077 as described above. Neutrophils were plated on cover slips as described above without additional stimulation. Use of SFs from patients was approved by the Institutional Review Board (IRB) of the University of Michigan (HUM00014692).

### DEK antibody purification from synovial fluids

SulfoLink Coupling Gel (Pierce Biotechnology, Rockford, IL, USA) was used to couple the recombinant human DEK protein and purify the DEK antibodies from four different JIA patients (de-identified). Briefly, 100 μg of the DEK protein was dissolved in 500 μl of coupling buffer (1 × NET buffer: 50 mM Tris, 150 mM NaCl, 5 mM EDTA, pH 8.5). It was coupled to 500 μl of washed SulfoLink gel (in a 10 ml chromatography column) by mixing with the resin for 20 min followed by 40 min incubation. Non-specific binding sites were blocked with 50 mM cysteine by mixing for 15 min followed by 30 min incubation. The column was washed with 16 × column volume of 1 M NaCl followed by another wash with distilled water. The column was equilibrated with 1 × NET buffer prior to the affinity purification step. All steps were performed at room temperature. Human SFs were adjusted to 10 mM Tris, pH 8.0 and centrifuged to remove any precipitates. A total of 3-5 ml of SFs was used at a time to purify DEK antibodies on the prepared column. The column was mixed and then rotated for at least 4 h (up to 16 h) at room temperature. The column was washed with 10 × column volume of 1 × NET buffer+0.5 M NaCl+0.5% NP-40, followed by a wash with 1 × NET buffer+0.5% NP-40, another wash of 1 × NET buffer, and a final wash of 0.1 × NET buffer. Antibody was eluted with 0.1 M glycine (pH 3.0) and neutralized with 1 M Tris (pH 8.0). These antibodies have been shown to be highly specific for recombinant DEK (ref. 5).

### Metamorph 7.7 for NET quantification

This program was created to quantify and compare NET to neutrophil ratios by the Center for Live Cell Imaging (CLCI) at the University of Michigan. The program takes two 24-bit (colour) images, a DAPI and a FITC stained image. The RGB channels are split and then the two green channels are added together, as well as the two blue channels. The minimum value is set to the average+standard deviation/2 of all the pixels in the image. This threshold is then turned into a binary, where it passes through image filters that connect some of the finer image structures, showing the presence of NETs. Regions are automatically created around the NETs, and information is pulled into an excel sheet indicating image number, plane number, region number, area, integrated green signal, nucleus count, green nets and nets/nucleus.

### Splenocytes preparation

Spleens were surgically removed from WT (3 males) and *Dek*-KO (3 males), placed into fresh RPMI 1640 medium and gently homogenized with the back of 10 ml syringe against a sterile, rough surface. Homogenized spleens were spun down in 50 ml RPMI medium at 1,200 rpm for 10 min at 4 °C and incubated for 3 min in 3 ml cold ACK lysis buffer (150 mM NH_4_Cl, 10 mM KHCO_3_, 0.1 mM Na_2_EDTA, pH=7.2-7.4). An additional 47 ml cold RPMI buffer was added to the lysate and spun down at 1,200 rpm for 10 min 4 °C (ref. 39).

### Flow cytometry

Splenocytes were purified as described above and were re-suspended in FACS buffer (1% FBS in PBS). TLR2 was detected by monoclonal anti-TLR2-FITC (Imegenex, IMG-6320C). Ficoll-purified bone marrow of WT control mice and *Dek-*KO mice or whole blood samples was re-suspended in 1% BSA and 1% horse serum in PBS. Samples were spun at 1,600 rpm for 5 min at 4 °C. Cell pellets were re-suspended with anti-Ly6G-FITC and or anti-CD11b–PE-Cy5 (BD Pharmingen, #553312) antibodies and incubated on ice for 30 min. Isotype-matched IgGs were used as negative control antibodies. Samples were centrifuged at 1,600 rpm for 5 min at 4 °C and fixed with 2% paraformaldehyde. Cell surface markers were analysed by FACS.

### H_2_O_2_ detection

Amplex Red reagent (Molecular Probes, Eugene, OR, USA) was used to colorimetrically determine levels of H_2_O_2_ secreted from peripheral blood and bone marrow neutrophils obtained from WT (3 females and 3 males) and *Dek*-KO (4 females and 2 males) mice. Specifically, 0.1 ml of 50 μM Amplex Red and 10 U ml^−1^ HRP, prepared in PBS, was added to each well of plated neutrophils (5 × 10^5^ per well in 96 well plates). Plates were incubated for an hour at 37 °C prior to quantifying H_2_O_2_ levels. Quantification was performed at a detection limit of 0.625 nM based on a standard curve generated from samples with known H_2_O_2_ concentrations. Samples were measured at Å560 nM using a Tecan GENios plate reader (Phenix, Australia)^40^.

### Peripheral murine neutrophil purification

Blood was obtained from WT (3 females and 3 males) and *Dek-*KO (4 females and 2 males) mice by cardiac puncture under terminal anaesthesia. Blood was collected into heparinized tubes (500 U per 1 ml blood). Cells were isolated by Histopaque-1083 in 1:1 ratio in 15 ml tubes. Neutrophils were recovered from the red blood cell (RBC) fraction in the bottom of the tube by 20% dextran solution (half the volume of the RBC), mixed, and RBCs allowed to sediment for 10 min at room temperature. Leukocyte-rich supernatant was collected from the top fraction and washed twice by 8 ml 0.2% BSA in PBS by centrifugation (1,500 rpm). Red blood cells were lysed with 2 ml RBC lysis buffer (BioLedgened) for 4 min on ice followed by 10 ml PBS wash. 1-2 × 10^6^ cells were plated on coverslips as described in the Experimental Procedures section in RPMI with 2% BSA and stimulated with 1 ng ml^−1^ PMA for 2 h (ref. 41).

### Western blot analysis

For cell signalling, bone marrow cells from WT (4 females and 5 males) and *Dek*-KO mice (4 females and 5 males) grown in 20% FBS and 30% L-cell supernatant (a mouse fibroblast cell line that secretes M-CSF) were isolated as described above. Macrophages were treated for 2 h prior stimulation with the proteasomes inhibitors MG-132 (1 μM) (EMD, Millipore). After stimulation with LPS and zymosan cells were harvested and lysed using 10 mM Hepes (pH 7.9), 1.5 mM MgCl, 10 mM KCl, and 0.1% NP40 in the presence of EDTA-free protease inhibitor. Equal amounts of proteins were loaded and analysed with the indicated antibodies by immunoblotting and as described above.

### Statistical analysis

Data are presented as means±s.e.m. The difference between means was analysed using the two-tail unpaired Student's *t*-test. A *P* value<0.05 was considered significant.

### Study approval

All protocols for animal and human studies were approved by the University of Michigan's Committee on Use and Care of Animal or the Institutional Review Board. Informed consent was obtained from all human subjects involved in the study.

### Data availability

The authors declare that the data supporting the findings of this study are available within the article and its Supplementary Information files, and from the corresponding author on request.

## Additional information

**How to cite this article:** Mor-Vaknin, N. *et al*. DEK-targeting DNA aptamers as therapeutics for inflammatory arthritis. *Nat. Commun.* **8,** 14252 doi: 10.1038/ncomms14252 (2017).

**Publisher's note:** Springer Nature remains neutral with regard to jurisdictional claims in published maps and institutional affiliations.

## Supplementary Material

Supplementary Information Supplementary Figures and Supplementary Tables.
