# Supplementary material for: DEK-targeting DNA aptamers as therapeutics for inflammatory arthritis
Source: Nat Commun. 2017 Feb 6;8:14252. doi: 10.1038/ncomms14252 (PMC5303823; doi:10.1038/ncomms14252)
Supplement: Supplementary Information — Supplementary Figures and Supplementary Tables. [file ncomms14252-s1.pdf]

A.

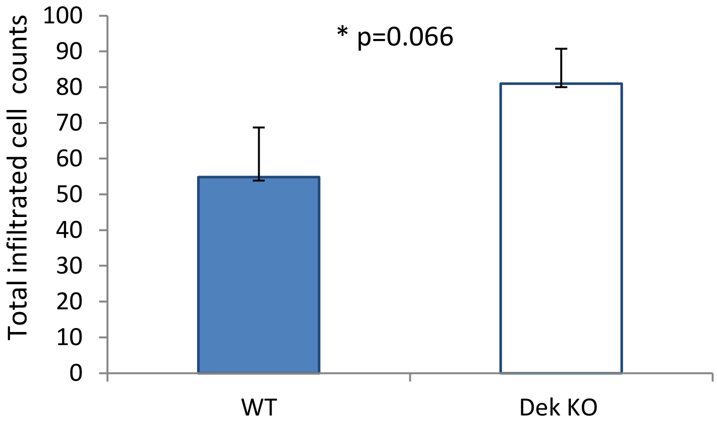

B.

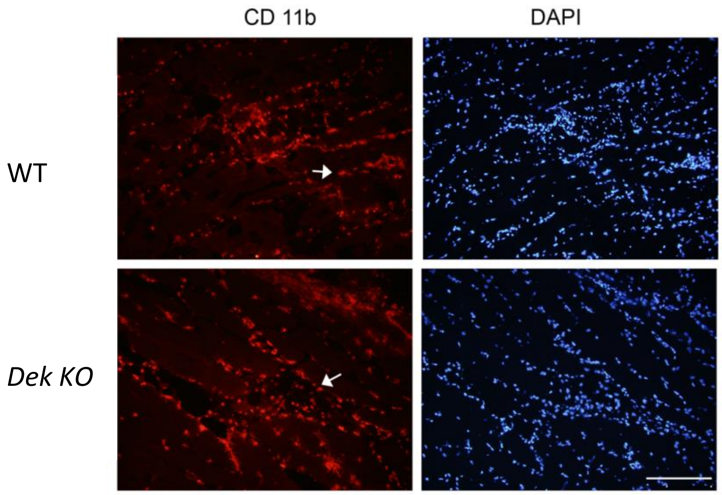

Supplementary Figure 1. Cell migration in response to zymosan-induced arthritis is the same in WT and *Dek* KO mice. Joint sections from WT (n=7) and *Dek* KO (n=11) zymosan-injected knees were analyzed for cell migration based on H&E slides assessed by 2 independent pathologists in a blind fashion. The difference between WT and *Dek* KO injected joints were not significate (p = 0.66), as determined by two-tailed unequal variance Student's t-test (error bars, s.e.m.). B. monocytes were detected by immunohistochemistry 24 hours after intra-articular

injection using the murine leukocyte/monocytic surface marker CD11b (in red): examples of positive cells are marked by arrows. Sections were also stained for cell nuclei with DAPI (blue). Magnification 20X, scale bar 200µm. *Dek* KO injected joints exhibit the same number of CD11b positive cells as do WT injected joints. Shown are representative images from 3 different sections from 3 WT and 3 *Dek* KO mice from two different independent experiments.

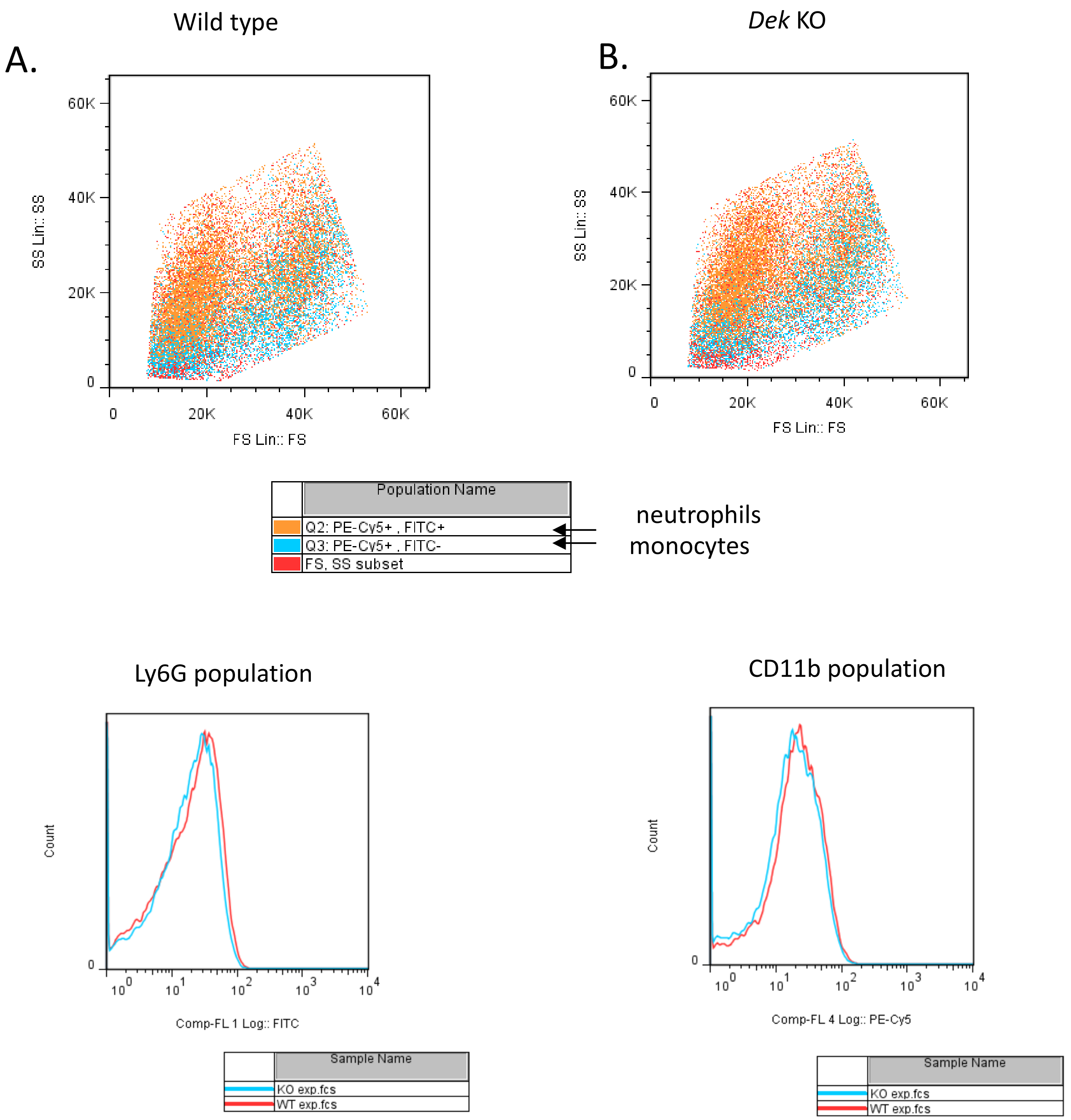
Wild type
Dek KO
A.
B.
SS Lin:: SS
FS Lin:: FS
0
20K
40K
60K
Population Name
Q2: PE-Cy5+ , FITC+
Q3: PE-Cy5+ , FITC-
FS, SS subset
neutrophils
monocytes
Ly6G population
CD11b population
Count
Comp-FL 1 Log:: FITC
Comp-FL 4 Log:: PE-Cy5
Sample Name
KO exp.fcs
WT exp.fcs

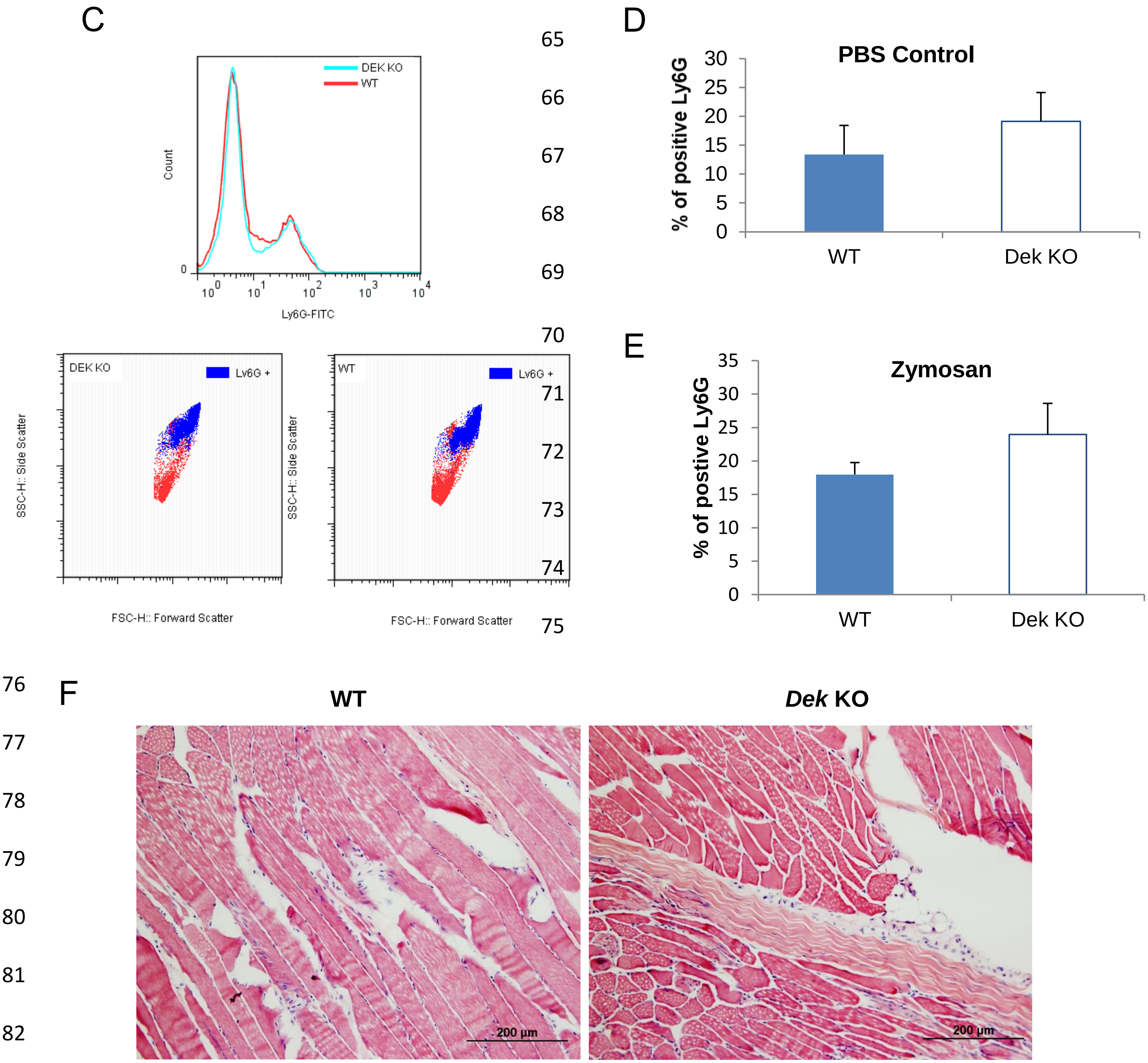

Supplementary Figure 2. Neutrophils from *Dek* KO mice are mature. Flow cytometry analysis of neutrophils purified from bone marrow of WT (A) and *Dek* KO mice (B) was performed using Ly6G–FITC (neutrophils) and CD11b-PE-Cy5 (found on neutrophils and monocytes). No difference in expression of Ly6G in *Dek* KO neutrophils was observed when

compared to WT as shown by the dot plots and histograms below. (C). Representative flow cytometry histogram of whole blood cells obtained from WT and *Dek* KO mice using Ly6G–FITC. No difference in expression of Ly6G was detected in *Dek* KO vs. WT peripheral blood. (D, E). Percentage of Ly6G positive cells in the peripheral blood of WT and *Dek* KO mice 24 hours after they received intra-articular injections with (D) PBS as a control or (E) zymosan, as calculated from 3 different individual WT or *Dek* KO mice (error bar, s.e.m.). (F). Representative H&E stained section from knee joints injected with PBS. Magnification 40X, scale bar 200µm. No significant difference was observed between WT (n=3) and *Dek* KO (n=3) joints and no signs of inflammation were detected by phathologists.

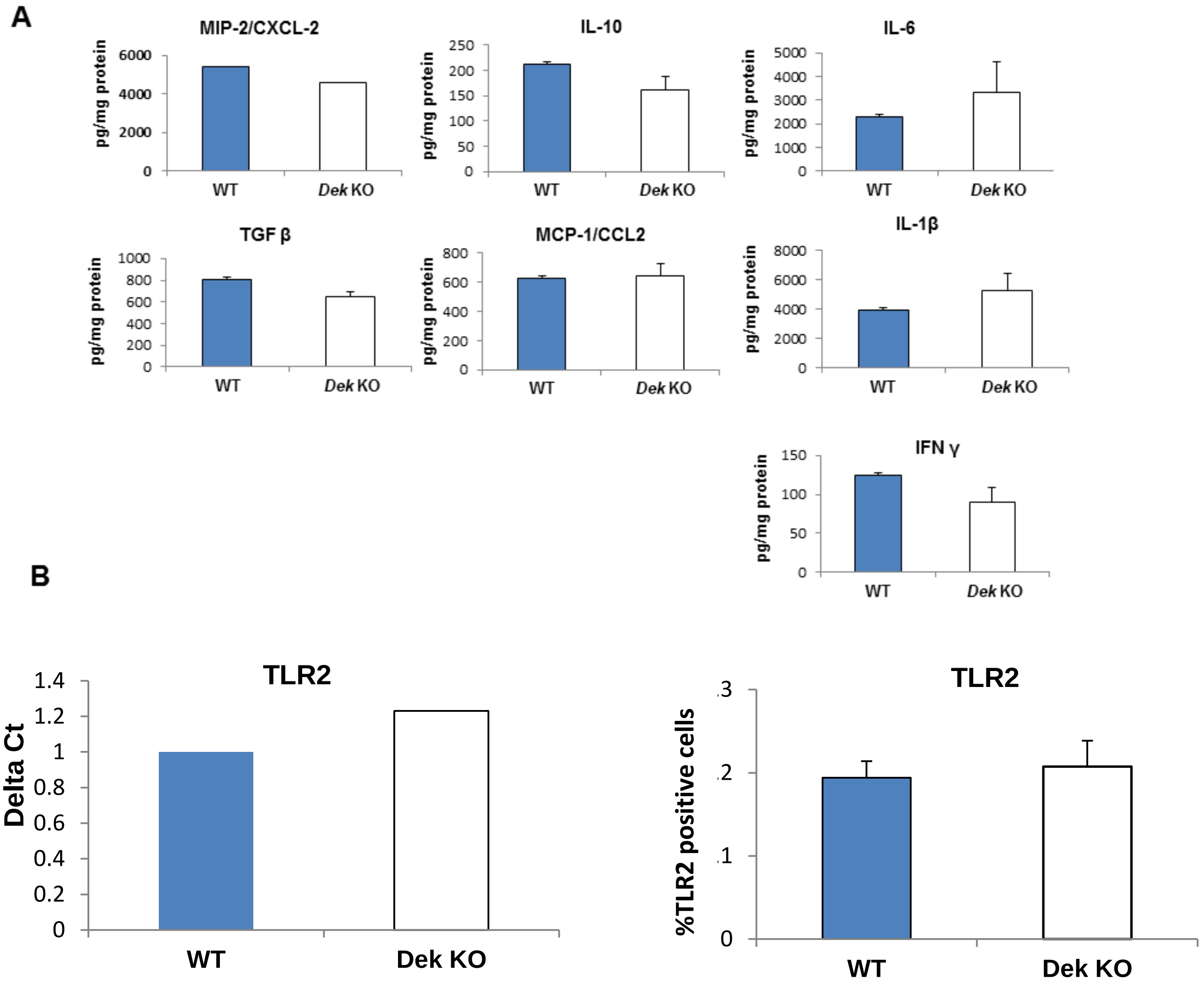

Supplementary Figure 3. No significant difference in expression of key pro-inflammatory cytokines and TLR2 in *Dek* KO vs. WT mice. (A). No significant differences were observed in IL-6, IL-1β, MIP-2, IL-10, IFN-γ, TGF-β and MCP-1 levels in knee homogenates of WT and *Dek* KO mice after zymosan injection as determined by two-tailed, unequal variance Student's t-test (error bars, s.e.m.). Cytokine levels were analyzed by ELISA and normalized by protein concentration. (B)**.** TLR2 levels of expression are similar in *Dek* KO and WT mice. cDNA was prepared from WT or *Dek* KO zymosan-injected knees and qPCR was used to determine TLR2

RNA levels, which showed no significant differences (left panel) as determined by two-tailed, unequal variance Student’s t-test (error bars, s.e.m.). TLR2 levels were also measured by flow cytometry of cells isolated from naïve WT and *Dek* KO spleens (right panel). Low levels of TLR2 were detected, but no difference in TLR2 expression was observed, consistent with the RNA data shown in the left panel.

A

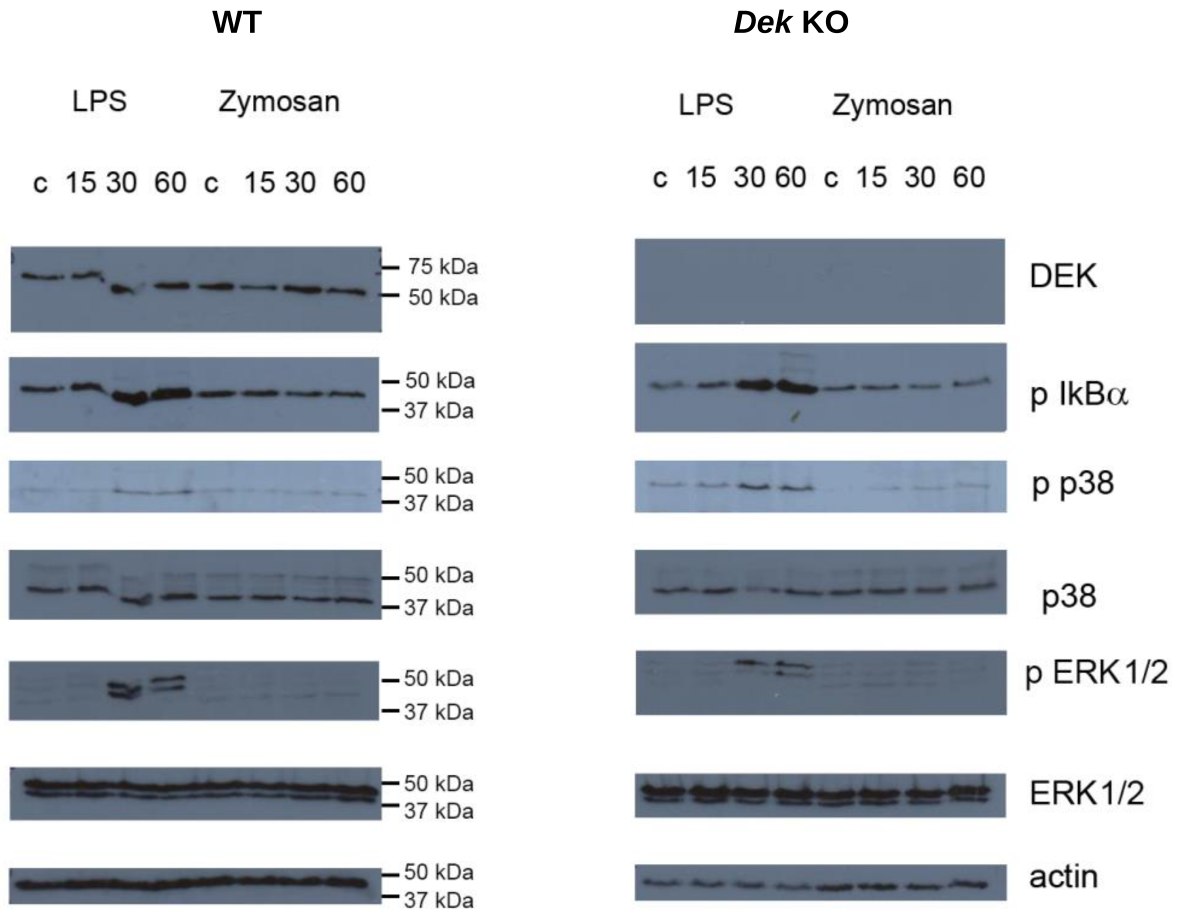

Supplementary Figure 4. Cell signaling profiles post-stimulation are unaltered in *Dek* KO cells. Bone marrow-derived macrophages collected from WT and *Dek* KO mice were stimulated with 1µg/mL LPS or zymosan for the indicated times. Western blot analysis of the cytosol fraction shows no significant differences in levels of phosphorylated IκB (p IκBα, (Ser 32)) or MAPK signaling (p38 and pERK1/2), which are downstream of IRAK1, in the absence of DEK. Results are representative of 3 different experiments.

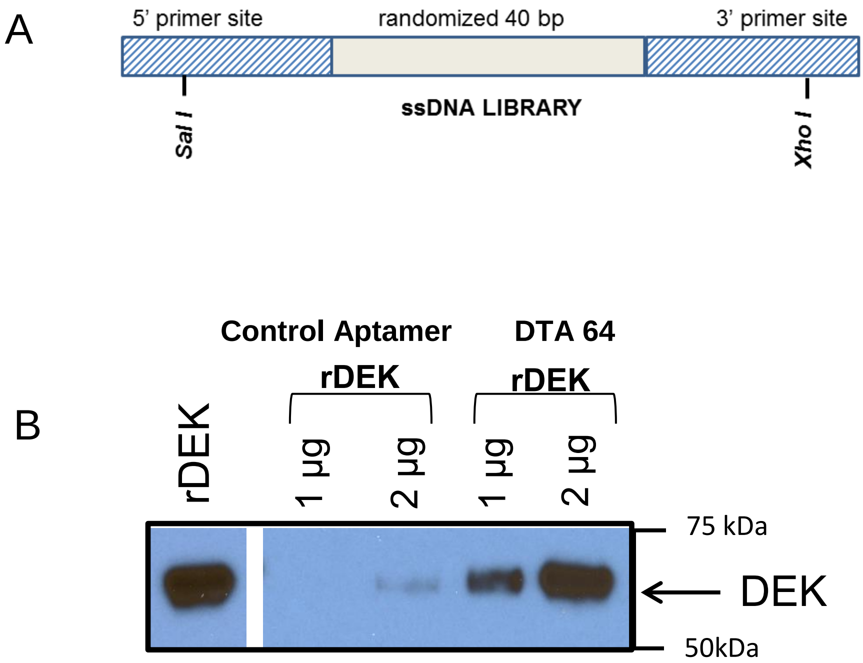

Supplementary Figure 5. Identifying DEK aptamers. (A). Recombinant DEK protein conjugated to nickel agarose beads was incubated with a library including a pool of 10^{14}-10^{16} random single-stranded DNA sequences. Each sequence includes 40 nucleotides (nt) flanked by 22 known nt on each side that will serve as primers for amplification by PCR. After extensive washes, the bound nt were eluted and amplified by PCR reaction. These steps were repeated up to 6 times to achieve more specificity. Nonspecific binding of the sequences to the naked beads was also eliminated. (B). To test aptamer-specific binding to DEK, we used two different concentrations (as indicated) of recombinant DEK protein incubated overnight at 4 ºC with magnetic beads conjugated to biotinylated control aptamer or anti-DEK aptamer (DTA 64). After incubation with recombinant DEK, the magnetic beads were washed 5 times with PBS and analyzed by immunoblot using a monoclonal antibody to DEK (BD Biosciences). Recombinant

DEK protein specifically binds to anti-DEK aptamers as compared to the scramble control aptamer in a concentration-dependent manner.

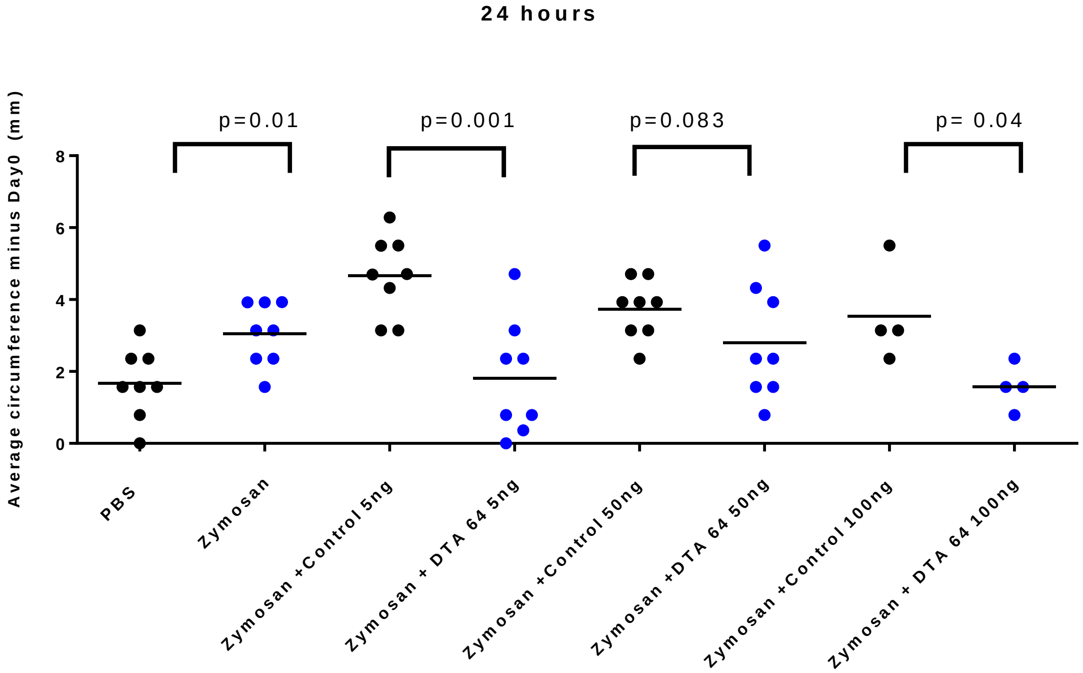

Supplementary Figure 6. Zymosan induction of joint inflammation is blocked by DEK aptamers. WT mice were injected on day 0 with 5 ng, 50 ng, or 100 ng per knee of non-specific DNA aptamer controls or DEK-specific aptamer (DTA-64) prior to injection with PBS alone or zymosan alone as negative and positive controls, respectively. Knee circumferences were measured at the time of injection (day 0), as well as at 24 hours following injection. Mean values of increased knee circumferences are shown at 24 hours after injection. Results shown are from 4-8 individual mice per group and from two independent experiments. Differences in the knee circumference between mice receiving control aptamers vs. DTA 64 aptamer (5-100 ng) were statistically significant as determined by the two tailed, unequal variance Student's t-test.

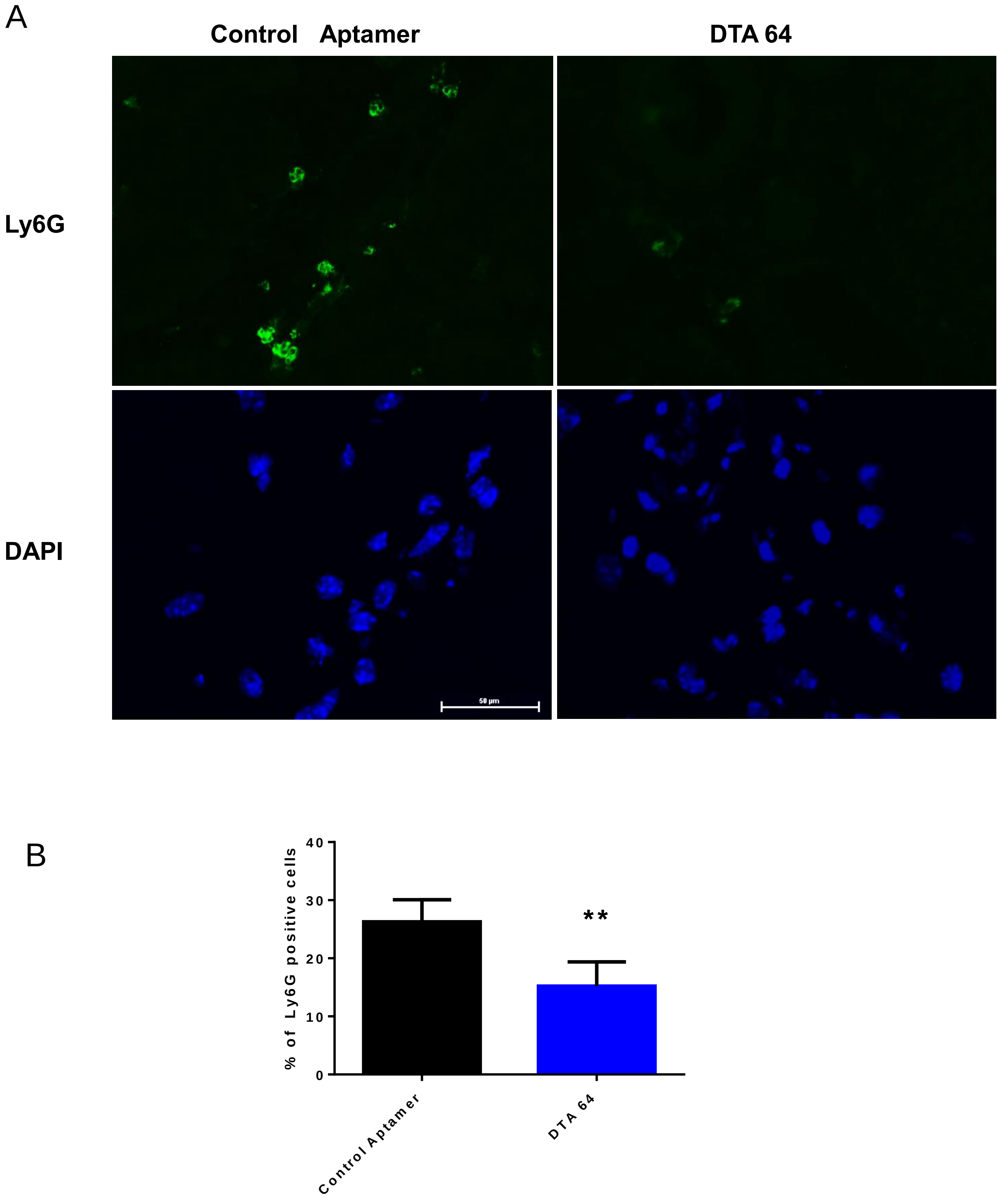
A
Control Aptamer
DTA 64
Ly6G
DAPI
50 µm
B
% of Ly6G positive cells
0
10
20
30
40
**
Control Aptamer
DTA 64

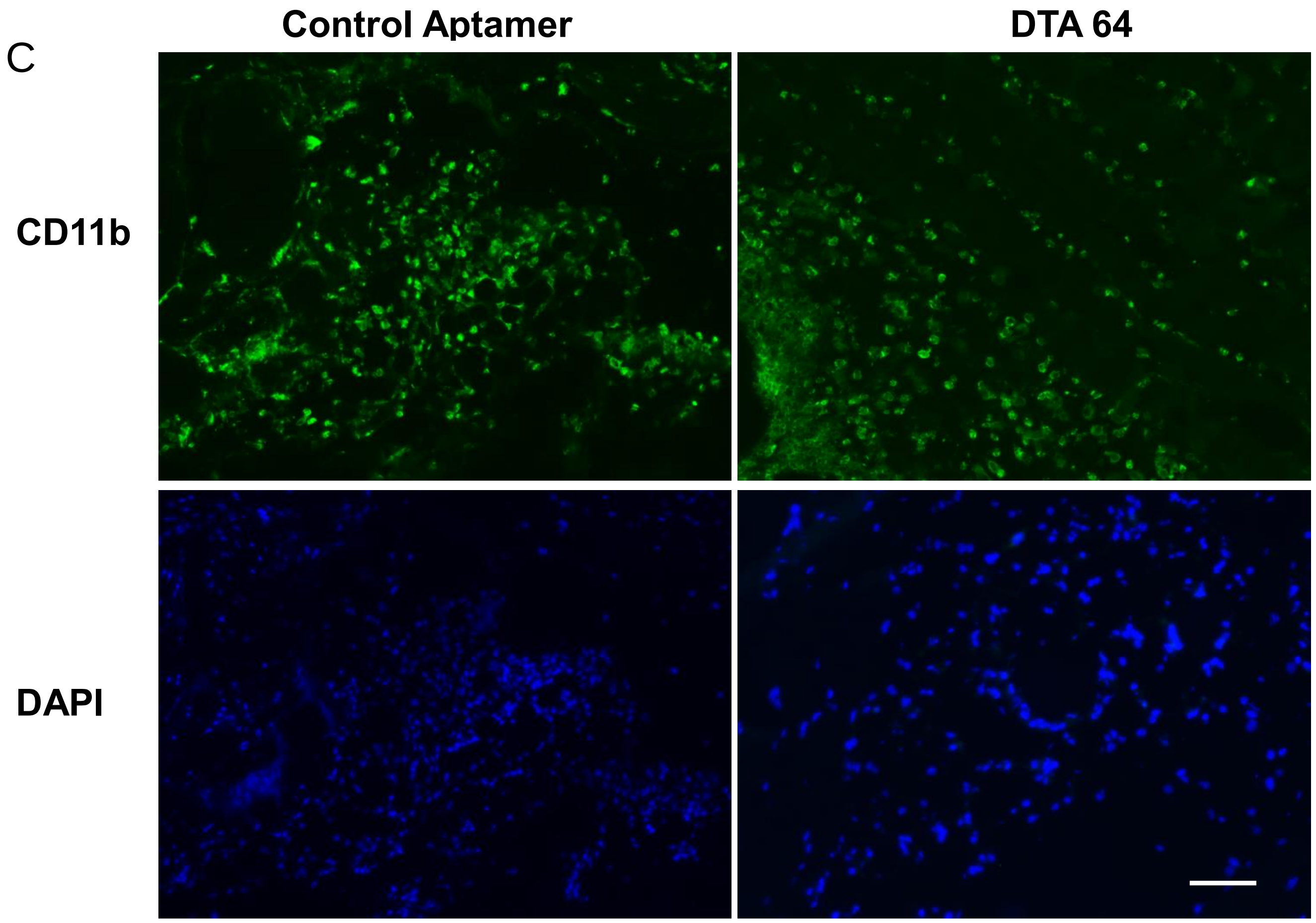
C
Control Aptamer
DTA 64
CD11b
DAPI

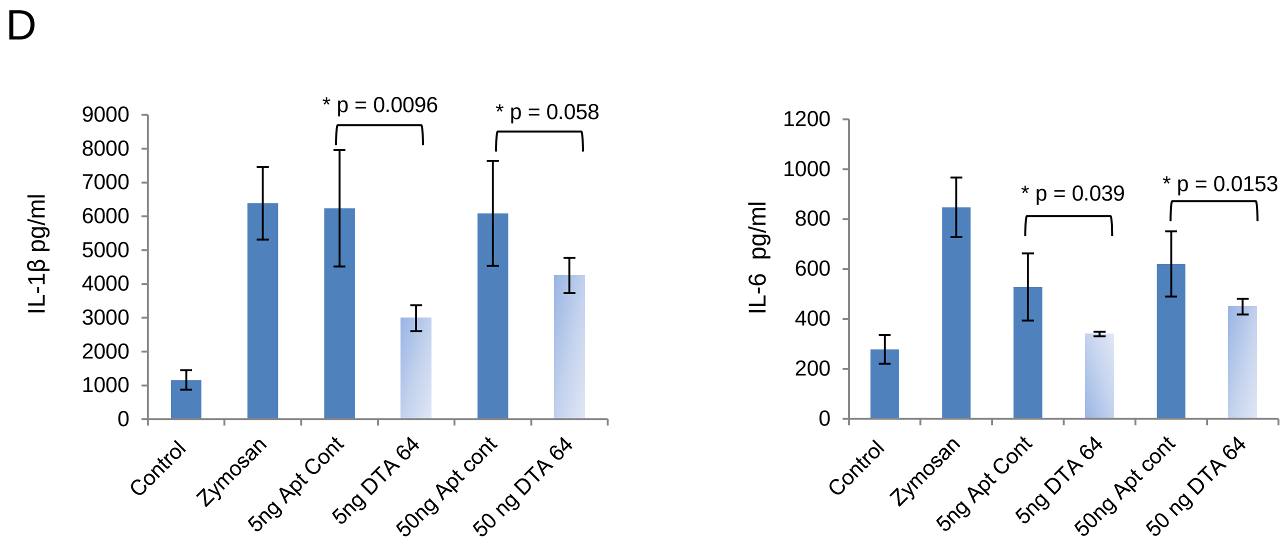
D
IL-1β pg/ml
9000
8000
7000
6000
5000
4000
3000
2000
1000
0
* p = 0.0096
* p = 0.058
Control
Zymosan
5ng Apt Cont
5ng DTA 64
50ng Apt cont
50 ng DTA 64
IL-6 pg/ml
1200
1000
800
600
400
200
0
* p = 0.039
* p = 0.0153
Control
Zymosan
5ng Apt Cont
5ng DTA 64
50ng Apt cont
50 ng DTA 64

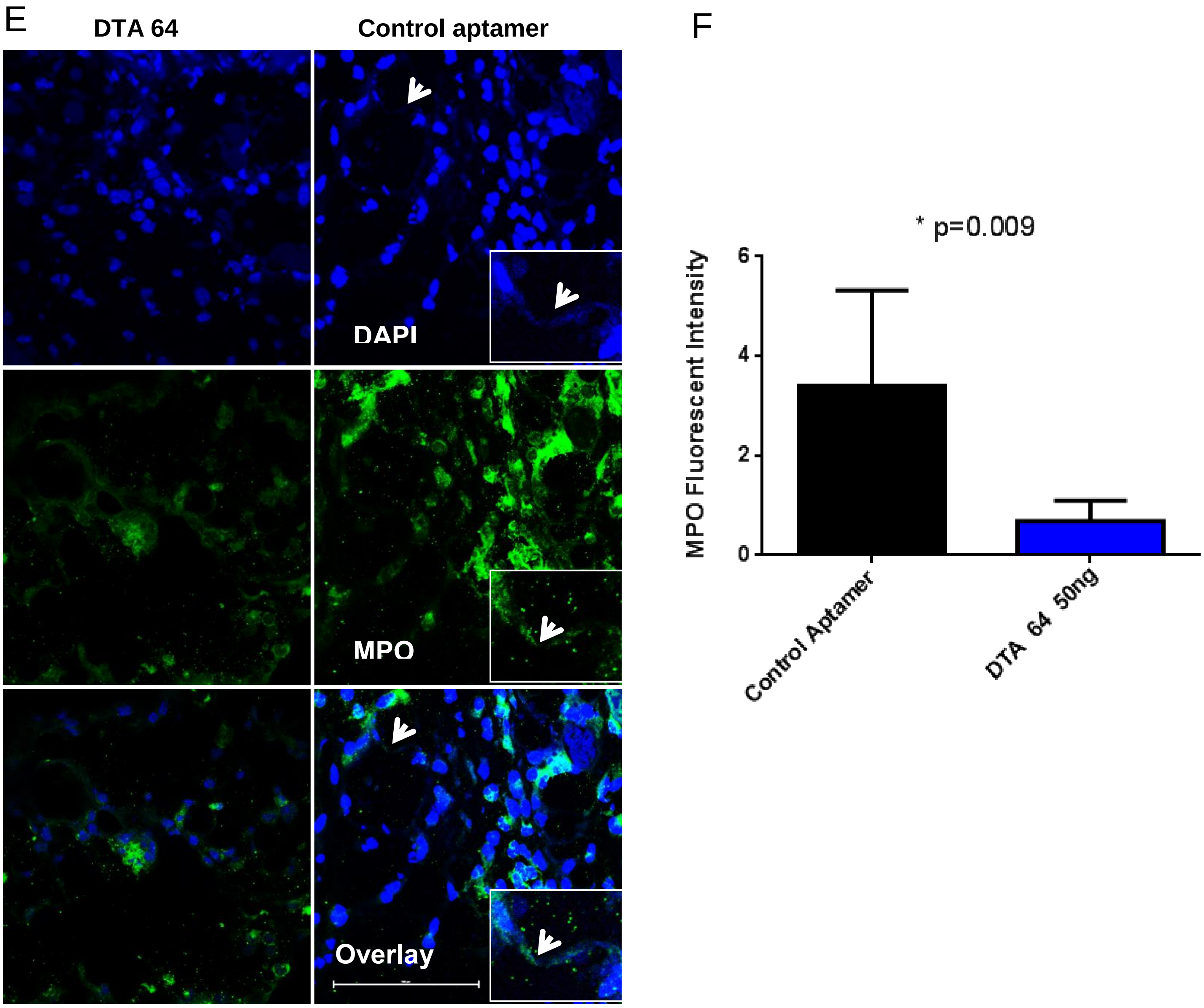
E
DTA 64
Control aptamer
DAPI
MPO
Overlay
F
* p=0.009
MPO Fluorescent Intensity
6
4
2
0
Control Aptamer
DTA 64 50ng

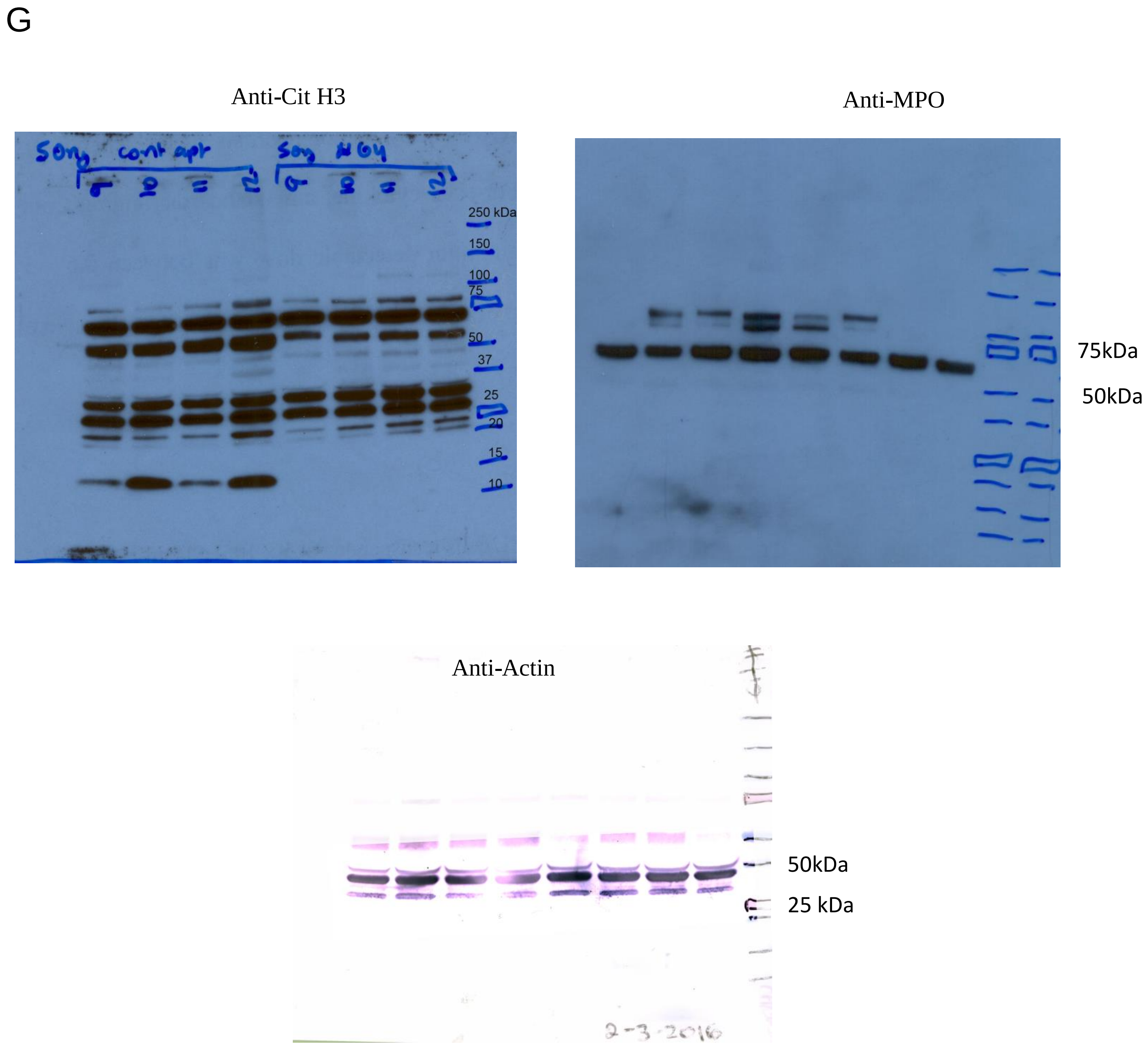

Supplementary Figure 7. DEK aptamers reduce neutrophil migration in response to zymosan. (A). Joint sections were analyzed for neutrophils by immunohistochemistry 48 hours after intra-articular injection of zymosan and either control (n=3) or anti-DEK aptamer (n=3) using the murine neutrophil surface marker Ly6-G (in green); cell nuclei are stained with DAPI (blue). Magnification 40X, scale bar 50µm. Images are representative of 3 different fields from 3 different mice as indicated above. (B). Joints injected with anti-DEK aptamers exhibit

significantly lower numbers of Ly6G positive cells as compared to joints injected with control aptamer. Results shown reflect the percentage of Ly6G positive cells from 5 different fields from each joint section from 3 different mice. *p = 0.031, as determined by two-tailed, unequal variance Student's t-test (error bars, s.e.m.). (C). Joint sections were analyzed for monocytes by immunohistochemistry 48 hours after intra-articular injection of zymosan and either control or anti-DEK aptamer using the monocyte surface marker CD11b (in green); cell nuclei are stained with DAPI (blue). No difference was observed in CD11b positive cells. Magnification 10X**, s**cale bar 100µm. Representative images of 3 control or anti-DEK aptamer and zymosan. The experiment was repeated twice. (D). Knee homogenates (n=3 to 4 mice/group) analyzed for a panel of pro-inflammatory cytokines (TNF-α, IL-10, IL-23, IFN-γ, TGF-β, RANTES, IL-13, MIP-2, MCP-1, IL-1α, IL-1β and IL-6) exhibited small but significant reductions only in IL-1β and IL-6 levels when comparing DTA-64 to control aptamer-treated groups, as determined by two-tailed, unequal variance Student's t-test (error bars, s.d. of two independent experiments). (E). Knee joint sections from mice receiving control aptamer or DTA 64 prior to intra-articular injection of zymosan were analyzed at 48 hours by immunohistochemistry using DAPI (blue) for DNA staining and the NET marker MPO (in green). Magnification 60X, scale bar 100µm (Nikon Confocal Microscope). Arrowheads indicate NETs. (F). Arbitrary fluorescent intensity of MPO staining analyzed for 10 fields of each section of 3 mice/group of zymosan/aptamer-injected knees significant difference (*p = 0.009) was found in MPO staining as determined by two-tailed, unequal variance Student's t-test (error bars, s.e.m.). Mean florescent intensity of the whole section was determined by Image J. (G). Full gel images for Figure 3B.

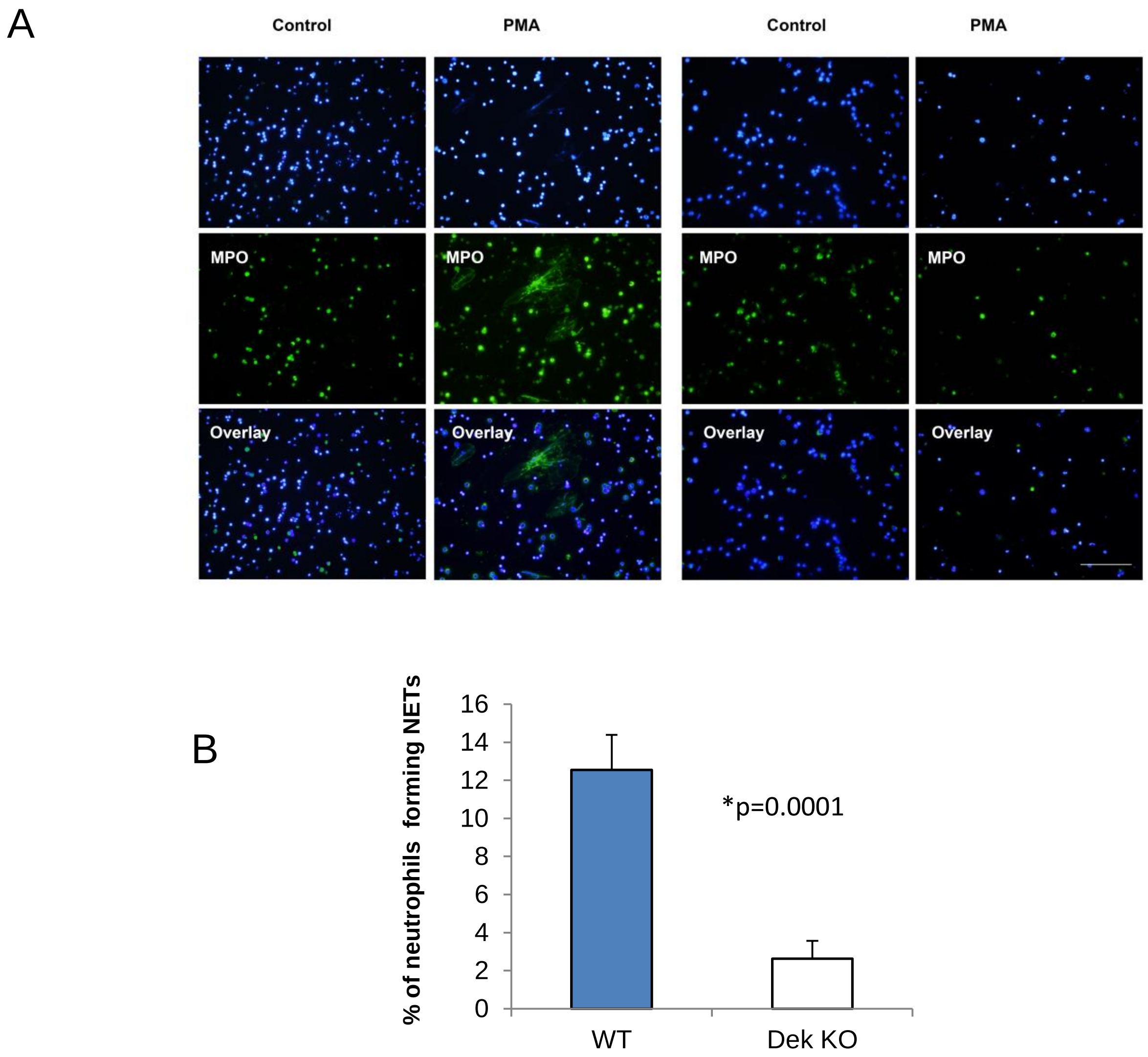

Supplementary Figure 8. Minimal NET formation is seen even after long-term stimulation of *Dek* KO neutrophils. (A). Neutrophils were purified from the bone marrow of WT and *Dek* KO mice as described in the Methods section. Neutrophils were stimulated with 1 ng mL^{-1} PMA for 8 hours and then fixed and stained with MPO (1:500, Dako) (green) and DAPI (blue). No formation of fully developed NETs was detected from the stimulated *Dek* KO cells. WT neutrophils readily made NETs. 40X magnification, scale bar 500μm. (B). The percentage of PMA-stimulated neutrophils with NETs seen in 10 different fields of WT and *Dek* KO cells as

counted by two independent individuals was significantly different (*p = 0.0001) as determined by two-tailed, unequal variance Student's t-test (error bars, s.e.m.).

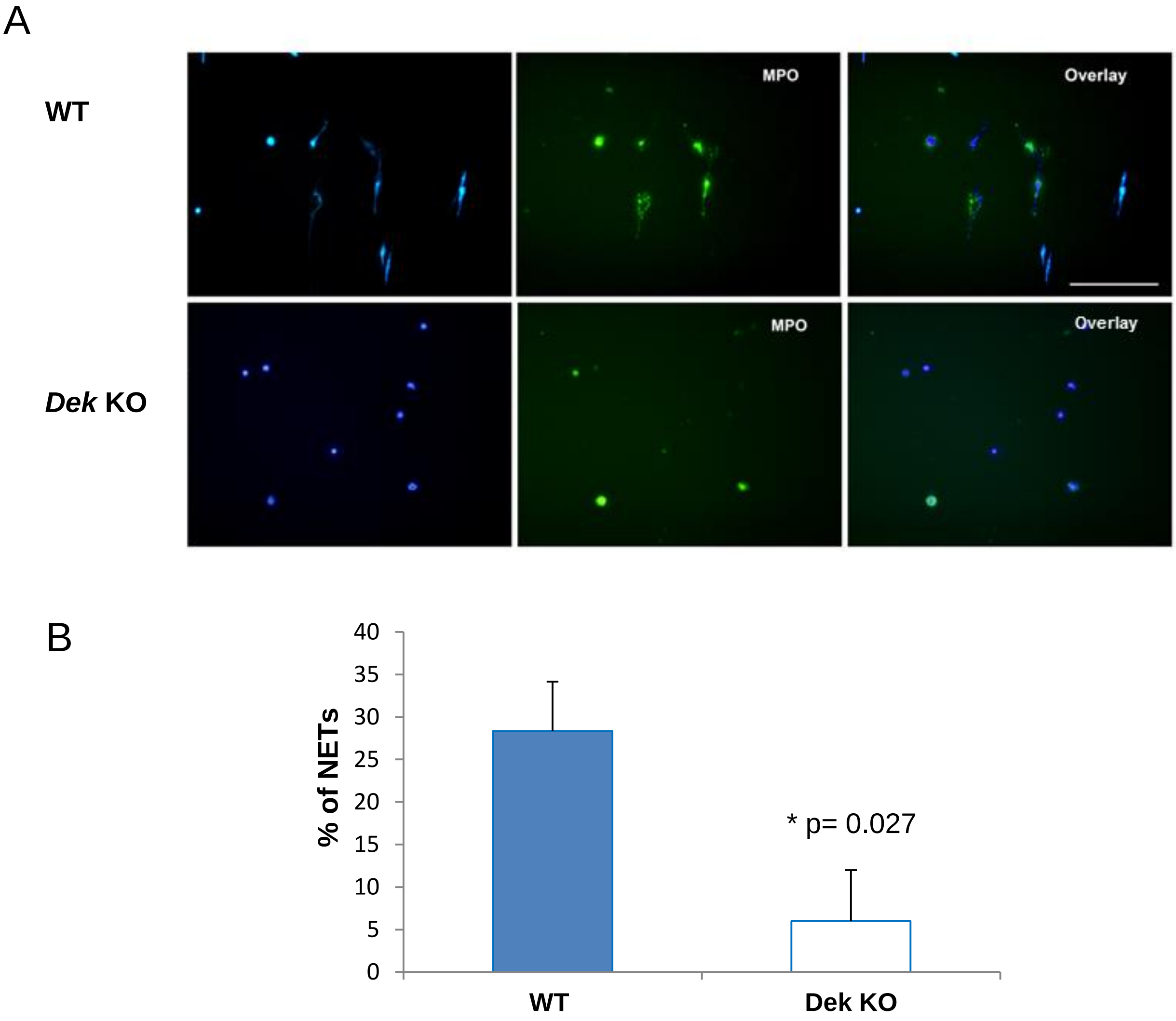

Supplementary Figure 9. Mouse peripheral blood neutrophils from *Dek* KO mice form markedly fewer NETs in response to stimulation than do peripheral blood neutrophils from WT mice. (A). Immunostaining of peripheral blood neutrophils purified from *Dek* KO and WT mice after 2 hour stimulation with 1 ng mL^{-1} PMA. Neutrophils were fixed and stained by MPO (green) and DAPI (blue). 40X magnification, scale bar 100µm. WT neutrophils show NET formation as expected after PMA stimulation, but minimal NET formation is detected in the stimulated *Dek* KO neutrophils as indicated by MPO and DAPI. (B). The percentage of

neutrophils that formed NETs after PMA stimulation in *Dek* KO vs. WT neutrophils was significantly different (*p = 0.027) as determined by two-tailed, unequal variance Student's t-test (error bars, s.e.m.). Results were calculated from 5 different fields of WT and *Dek* KO peripheral blood neutrophils.

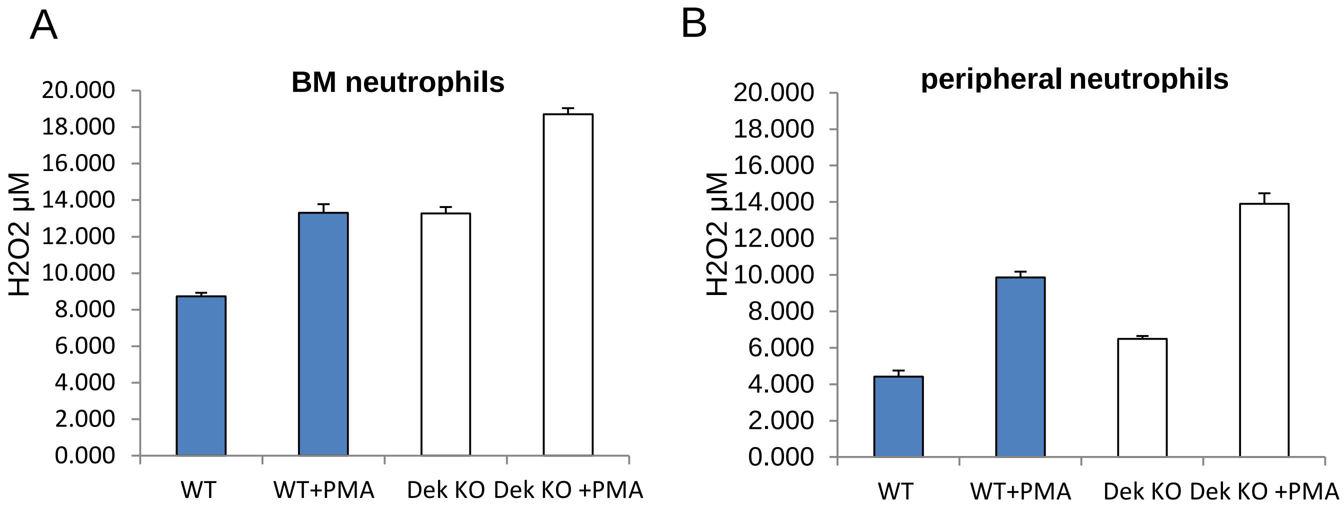

Supplementary Figure 10. Peripheral and BM neutrophils from *Dek* KO mice produce ROS to the same extent as do those from WT mice before and after PMA stimulation. (A). BM or (B). peripheral blood neutrophils obtained from *Dek* KO and WT mice were incubated with or without PMA (1 ng mL^{-1}) prior to determining H_2O_2 concentration in the supernatants. Data shown represent 2 independent experiments performed in triplicate and the significance was determined by two-tailed, unequal variance Student's t-test (error bars, s.e.m.).

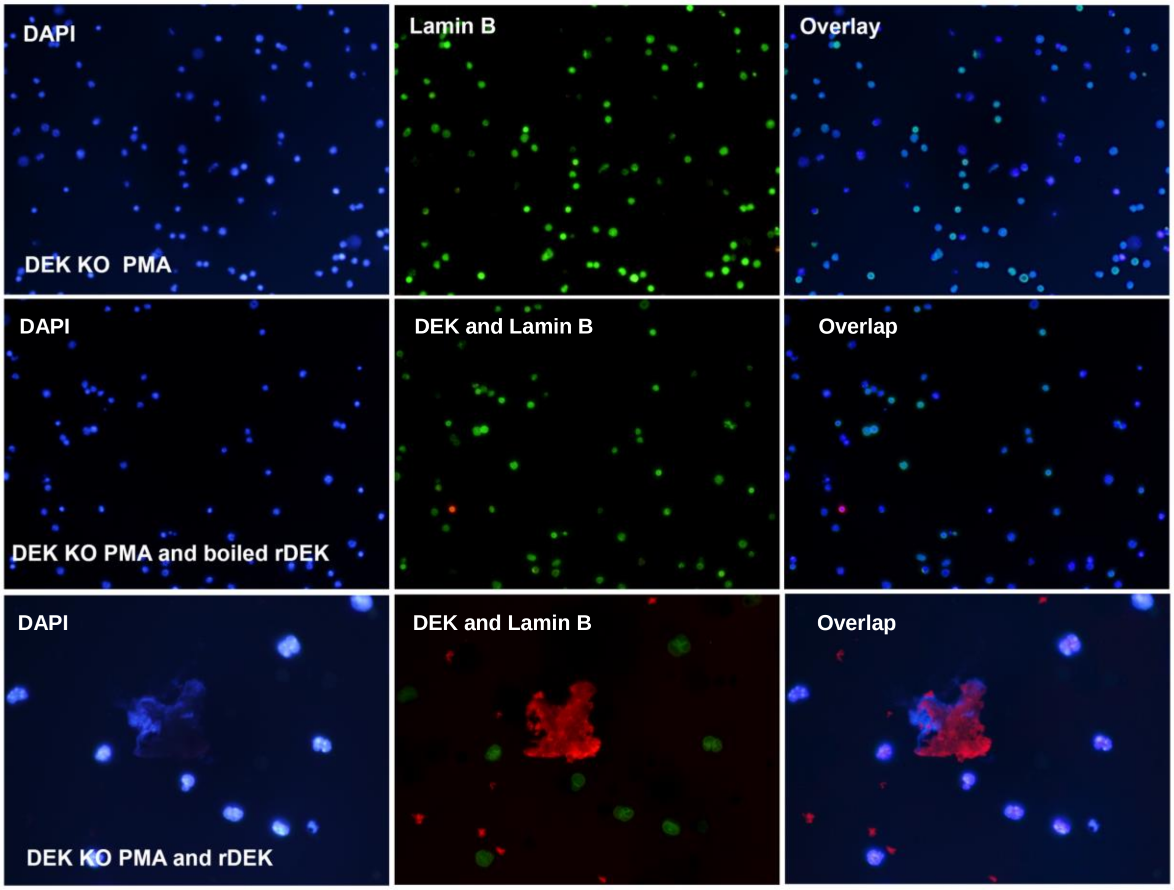

Supplementary Figure 11. Bioactive DEK is needed to restore NET formation by *Dek* KO neutrophils. *Dek* KO neutrophils treated with PMA alone (upper panel), boiled recombinant DEK (middle panel, 20X magnification) or native recombinant DEK (lower panel, 40X magnification, scale bar 100µm) prior to PMA stimulation. Cells were fixed and stained for DEK (Red) and Lamin B (Green). NETs were observed only with the native recombinant DEK, further confirming the specific role of biologically competent DEK in NET biology.

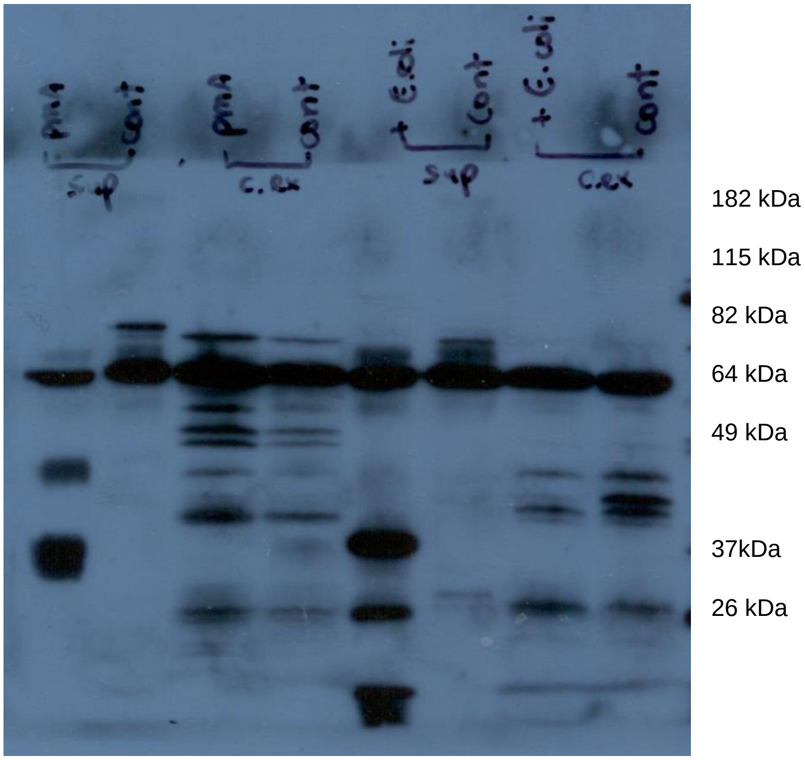

Supplementary Figure 12. DEK is released into the extracellular space by human neutrophils. Original gel for Figures 6 A and B.

A

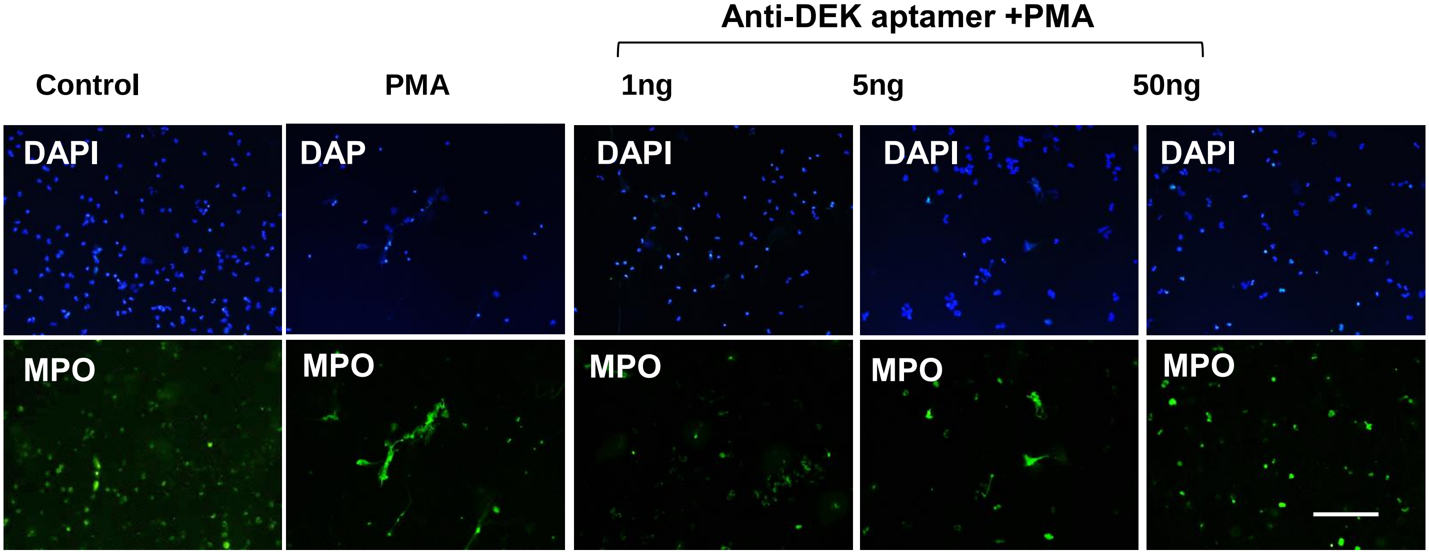

B

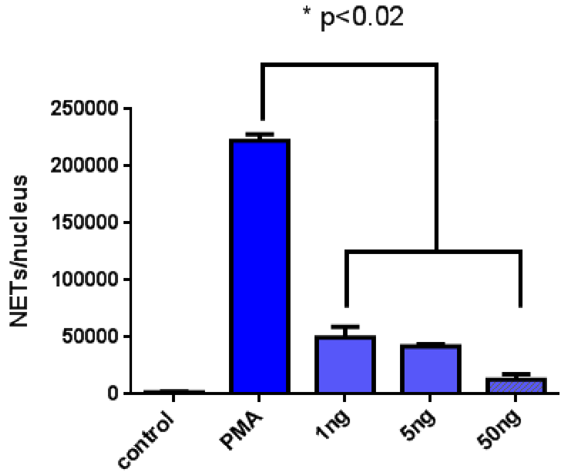

Supplementary Figure 13. DEK aptamers block NET formation by activated human neutrophils. (A). Peripheral blood human neutrophils were obtained from healthy control individuals and plated on glass coverslips. Aptamers to DEK were added at 1-50 ng to the neutrophil culture prior to PMA stimulation (10 ng mL^{-1}). Neutrophils were incubated for 1 hour at 37° C, then fixed and stained for NETs with anti-MPO antibodies (green) and DAPI for DNA (blue) (20X magnification, scale bar 200µm). The same results were obtained with a second

DEK aptamer (not shown). (B). NET staining intensity determined by Metamorph 7.7. demonstrates significant reduction in NET formation after treatment with anti-DEK aptamer ($p < 0.02$ as determined by two-tailed, are representative of at least 3 different independent experiments using neutrophils from 3 different donors.

Supplementary Table:

Table 1.The asymmetric PCR (repeated unidirectional primer extension) components were as follows:

**Reagents**	**Amount per reaction (uL)**
DNA from Symmetric PCR	10
Taq Buffer 10x	10
dNTPs (8mM)	2.5
5' LIB SEL No T7 (non-radiolabeled)	0.25
^{32}p 5'	1,000,000 CPM
Taq DNA pol (1:20 dilution of stock)	5
H_2O	Up to 100 uL

The asymmetric PCR conditions were: 94 ºC 5", [94 ºC 30", 59 ºC 0", 72 ºC 30"] x15 rounds, 72 ºC 7", 4 ºC hold.

Table 2. Inflammatory response and autoimmune PCR array (QIAGEN/SABiocience PAMM-0077A-2) of knee homogenates from zymosan injected joints comparing *Dek* KO to WT.

Symbol	Well	AVG ΔC_t (Ct(GOI) - Ave Ct (HKG))		$2^{\wedge}\text{-}\Delta C_t$		Fold Change	Fold Up- or Down-Regulation
		Test Sample	Control Sample	Test Sample	Control Sample	Test Sample /Control Sample	Test Sample /Control Sample
Bcl6	A01	2.16	2.06	0.22375627	0.23981603	0.93	-1.07
C3	A02	-0.74	0.46	1.67017584	0.72698626	2.30	2.30
C3ar1	A03	5.76	5.26	0.01845301	0.0260965	0.71	-1.41
C4b	A04	3.96	3.66	0.06425711	0.07910979	0.81	-1.23
Ccl1	A05	12.26	12.76	0.00020388	0.00014416	1.41	1.41
Ccl11	A06	3.96	5.26	0.06425711	0.0260965	2.46	2.46
Ccl12	A07	4.56	4.46	0.04239389	0.04543664	0.93	-1.07
Ccl17	A08	12.26	12.76	0.00020388	0.00014416	1.41	1.41
Ccl19	A09	1.26	2.16	0.41754396	0.22375627	1.87	1.87
Ccl2	A10	0.06	0.16	0.95926412	0.89502507	1.07	1.07
Ccl20	A11	12.26	12.76	0.00020388	0.00014416	1.41	1.41
Ccl22	A12	12.26	12.76	0.00020388	0.00014416	1.41	1.41
Ccl24	B01	12.26	12.76	0.00020388	0.00014416	1.41	1.41
Ccl25	B02	8.26	8.36	0.00326206	0.00304361	1.07	1.07
Ccl3	B03	4.36	5.66	0.04869779	0.01977745	2.46	2.46
Ccl4	B04	0.66	1.26	0.6328783	0.41754396	1.52	1.52
Ccl5	B05	5.56	5.16	0.02119694	0.02796953	0.76	-1.32
Ccl7	B06	0.76	0.46	0.59049633	0.72698626	0.81	-1.23
Ccl8	B07	5.96	7.46	0.01606428	0.00567958	2.83	2.83
Ccr1	B08	1.46	2.26	0.36349313	0.20877198	1.74	1.74
Ccr2	B09	5.36	4.96	0.02434889	0.03212856	0.76	-1.32
Ccr3	B10	4.96	5.66	0.03212856	0.01977745	1.62	1.62
Ccr4	B11	12.26	12.76	0.00020388	0.00014416	1.41	1.41
Ccr7	B12	12.26	12.76	0.00020388	0.00014416	1.41	1.41
Cd40	C01	9.46	8.76	0.0014199	0.00230663	0.62	-1.62
Cd40lg	C02	11.06	11.36	0.00046839	0.00038045	1.23	1.23
Cebpb	C03	-2.44	-2.34	5.42641731	5.06302638	1.07	1.07
Crp	C04	12.26	12.76	0.00020388	0.00014416	1.41	1.41
Csf1	C05	2.26	2.36	0.20877198	0.19479114	1.07	1.07
Cxcl1	C06	4.46	5.56	0.04543664	0.02119694	2.14	2.14

Cxcl10	C07	5.46	4.46	0.02271832	0.04543664	0.50	-2.00
Cxcl11	C08	11.06	8.36	0.00046839	0.00304361	0.15	-6.50
Cxcl2	C09	-2.14	-1.14	4.40762046	2.20381023	2.00	2.00
Cxcl3	C10	1.26	2.26	0.41754396	0.20877198	2.00	2.00
Cxcl5	C11	0.36	0.46	0.77916458	0.72698626	1.07	1.07
Cxcl9	C12	5.16	5.16	0.02796953	0.02796953	1.00	-1.00
Cxcr4	D01	3.46	3.96	0.09087328	0.06425711	1.41	1.41
Fasl	D02	12.26	12.76	0.00020388	0.00014416	1.41	1.41
Flt3l	D03	5.56	5.96	0.02119694	0.01606428	1.32	1.32
Fos	D04	3.26	3.36	0.10438599	0.09739557	1.07	1.07
Hdac4	D05	3.96	3.76	0.06425711	0.07381204	0.87	-1.15
Ifng	D06	12.26	12.76	0.00020388	0.00014416	1.41	1.41
Il10	D07	9.16	9.96	0.0017481	0.00100402	1.74	1.74
Il10rb	D08	2.26	1.96	0.20877198	0.25702846	0.81	-1.23
Il18	D09	5.86	5.26	0.01721727	0.0260965	0.66	-1.52
Il18rap	D10	12.26	12.76	0.00020388	0.00014416	1.41	1.41
Il1a	D11	3.26	4.66	0.10438599	0.03955489	2.64	2.64
Il1b	D12	-0.54	-0.14	1.45397252	1.10190512	1.32	1.32
Il1f10	E01	12.26	12.76	0.00020388	0.00014416	1.41	1.41
Il1r1	E02	3.06	2.16	0.11990801	0.22375627	0.54	-1.87
Il1rap	E03	5.06	5.16	0.029977	0.02796953	1.07	1.07
Il1rn	E04	4.86	5.16	0.03443453	0.02796953	1.23	1.23
Il22	E05	12.26	12.76	0.00020388	0.00014416	1.41	1.41
Il22ra2	E06	9.36	9.26	0.00152181	0.00163103	0.93	-1.07
Il23a	E07	10.76	11.96	0.00057666	0.000251	2.30	2.30
Il23r	E08	12.26	12.06	0.00020388	0.0002342	0.87	-1.15
Il6	E09	3.16	3.56	0.11187813	0.08478777	1.32	1.32
Il6ra	E10	3.76	3.86	0.07381204	0.06886907	1.07	1.07
Il7	E11	12.26	12.76	0.00020388	0.00014416	1.41	1.41
Cxcr1	E12	9.26	9.46	0.00163103	0.0014199	1.15	1.15
Cxcr2	F01	3.06	3.26	0.11990801	0.10438599	1.15	1.15
Il9	F02	12.26	12.76	0.00020388	0.00014416	1.41	1.41
Itgb2	F03	2.36	2.46	0.19479114	0.18174656	1.07	1.07
Kng1	F04	12.26	12.76	0.00020388	0.00014416	1.41	1.41
Lta	F05	10.46	11.96	0.00070995	0.000251	2.83	2.83
Ltb	F06	5.66	5.36	0.01977745	0.02434889	0.81	-1.23
Ly96	F07	6.06	6.66	0.0149885	0.00988872	1.52	1.52
Myd88	F08	5.26	5.76	0.0260965	0.01845301	1.41	1.41
Nfatc3	F09	3.96	3.46	0.06425711	0.09087328	0.71	-1.41
Nfkb1	F10	4.26	4.26	0.05219299	0.05219299	1.00	-1.00
Nos2	F11	2.36	5.56	0.19479114	0.02119694	9.19	9.19

Nr3c1	F12	1.86	1.26	0.27547628	0.41754396	0.66	-1.52
Ripk2	G01	7.16	7.46	0.00699238	0.00567958	1.23	1.23
Tirap	G02	5.36	5.16	0.02434889	0.02796953	0.87	-1.15
Tlr1	G03	7.96	9.56	0.00401607	0.00132481	3.03	3.03
Tlr2	G04	4.86	5.16	0.03443453	0.02796953	1.23	1.23
Tlr3	G05	7.76	8.76	0.00461325	0.00230663	2.00	2.00
Tlr4	G06	3.56	4.06	0.08478777	0.05995401	1.41	1.41
Tlr5	G07	8.36	8.46	0.00304361	0.00283979	1.07	1.07
Tlr6	G08	5.96	6.86	0.01606428	0.00860863	1.87	1.87
Tlr7	G09	10.16	9.96	0.00087405	0.00100402	0.87	-1.15
Tnf	G10	6.16	7.16	0.01398477	0.00699238	2.00	2.00
Tnfsf14	G11	9.26	7.76	0.00163103	0.00461325	0.35	-2.83
Tollip	G12	4.26	3.86	0.05219299	0.06886907	0.76	-1.32
Gusb	H01	4.86	4.86	0.03443453	0.03443453	1.00	-1.00
Hprt	H02	2.26	2.16	0.20877198	0.22375627	0.93	-1.07
Hsp90ab1	H03	-1.14	-1.54	2.20381023	2.90794503	0.76	-1.32
Gapdh	H04	-3.44	-3.24	10.8528346	9.44794129	1.15	1.15
Actb	H05	-2.54	-2.24	5.81589007	4.72397065	1.23	1.23

Fold-Change (2^(- Delta Delta Ct)) is the normalized gene expression (2^(- Delta Ct)) in the Test Sample divided by the normalized gene expression (2^(- Delta Ct)) in the Control Sample.

Fold-Regulation represents fold-change (*Dek* KO vs. WT) results in a biologically meaningful way. Fold-change values greater than one indicates a positive- or an up-regulation in *Dek* KO sample vs. WT. In this scenario, the fold-regulation is equal to the fold-change. Fold-change values less than one indicate a negative or down-regulation. In this scenario, the fold-regulation is the negative inverse of the fold-change.
